# Management of BMI Is a Potential New Approach for the Prevention of Idiopathic Pulmonary Fibrosis

**DOI:** 10.3389/fgene.2022.821029

**Published:** 2022-03-11

**Authors:** Yuchao Ma, Chang Feng, Haibo Tang, Peizhi Deng, Yalan Li, Jie Wang, Shaihong Zhu, Liyong Zhu

**Affiliations:** ^1^ Department of Cardiothoracic Surgery, The Third Xiangya Hospital, Central South University, Changsha, China; ^2^ Department of Oncology, Xiangya Cancer Center, Xiangya Hospital, Central South University, Changsha, China; ^3^ Clinical Research Center, The Third Xiangya Hospital, Central South University, Changsha, China; ^4^ Department of Metabolic and Bariatric Surgery, The Third Xiangya Hospital, Central South University, Changsha, China

**Keywords:** Mendelian randomization, obesity, body mass index, idiopathic pulmonary fibrosis, causal inference

## Abstract

**Aims:** Current idiopathic pulmonary fibrosis (IPF) therapies usually show a poor outcome or treatment efficacy. The search for new risk factors has significant implications in preventing, delaying, and treating IPF. The association between obesity and the risk of IPF is not clear. This study aimed to investigate the role of different obesity types in IPF risk, which provides the possibility of weight loss as a new approach for IPF prevention.

**Methods:** We conducted a two-sample Mendelian randomization (MR) analysis to assess the causal effect of obesity on IPF risk. We collected summary data of genetically determined obesity-related traits, including body mass index (BMI), waist circumference (WC), and waist-to-hip ratio (WHR) from large-scale consortia (the sample size ranging from 232,101 to 681,275), and genetic association with IPF from one of the largest meta-analyses including 2,668 cases. A total of 35–469 single nucleotide polymorphisms were selected as instrumental variables for obesity-related traits. We further performed multivariable MR to estimate the independent effect of BMI and WC on the risk of IPF.

**Results:** Increased BMI and WC were associated with higher risk of IPF [odds ratio (OR) = 1.51, 95% confidence interval (CI) (1.22–1.87), *p* = 1.27 × 10^–4^, and OR = 1.71, 95% CI (1.08–2.72), *p* = 2.33 × 10^–2^, respectively]. Similar results for the BMI and WC were obtained in the replicated analysis. Subsequently, only the result for BMI survived following the multiple testing correction and showed good consistency with the weighted median estimator. Sensitivity analyses indicated that there was no heterogeneity or horizontal pleiotropy for MR estimations. Further multivariable MR suggested that the BMI showed the same direction and similar magnitude with that in the univariable MR analysis. There was little evidence to support the causal role of WHR on the risk of IPF in this study.

**Conclusion:** Genetically determined BMI demonstrates a causal risk for IPF, which offers a novel insight into probing potential mechanisms. Meanwhile, these results also suggest that weight loss may be beneficial to IPF prevention.

## 1 Introduction

Idiopathic pulmonary fibrosis (IPF), a kind of idiopathic interstitial pneumonia with diffuse parenchymal abnormalities and lesions, is the most frequent and severe form of fibrotic lung disease (John et al., 2020). The incidence of IPF has risen over time, typically affecting patients over 65 years with a median survival time of 2–4 years from diagnosis (Ley et al., 2011; Raghu et al., 2014; Esposito et al., 2015). However, the precise factors that initiate the process of IPF are still unknown. IPF is now generally considered as a consequence of environmental risk factors, including smoking (Baumgartner et al., 1997; Taskar and Coultas, 2006), viral infection (Naik and Moore, 2010), metal and wood dusts, agriculture and farming (Taskar and Coultas, 2006; Raghu et al., 2011), and interacting with genetic susceptibility (Evans et al., 2016). One possible explanation is that nonspecific injury to the epithelial barrier and pulmonary parenchyma by these risk factors initiates the disease process of IPF in susceptible individuals. Nevertheless, these risk factors do not seem to explain very much of the progressive nature of IPF or the higher risk of pulmonary fibrosis with age. There may be many more factors that remain undiscovered. Therefore, broadening our understanding of the risk factors for IPF is of tremendous significance to its prevention, retardation, and treatment.

In a phase III trial of pirfenidone in 1,247 patients with IPF, the proportion of patients with obesity was 44% (Glassberg et al., 2019), suggesting that obesity may play an essential role in IPF. Of note, given this cross-sectional result, we cannot identify the temporal relationship between obesity and IPF due to potential confounders and reverse causation. By contrast, Mendelian randomization (MR) offers an opportunity to reliably assess the causal effects between obesity types and IPF risk.

MR is considered as “nature's randomized control trial” (Thanassoulis and O’Donnell, 2009), using genetic variants to proxy the exposures of interest and explore the causal relationships with outcomes (Smith and Ebrahim, 2003), which is similar to different random interventions in randomized controlled trials. Thus, in our study, we performed a two-sample MR study to evaluate the effect magnitude and direction of obesity on the risk of IPF and estimate the independent effect of body mass index (BMI), waist circumference (WC), and waist-to-hip ratio (WHR) using multivariable MR analysis.

## 2 Materials and Methods

### 2.1 Study Design

**FIGURE 1:**
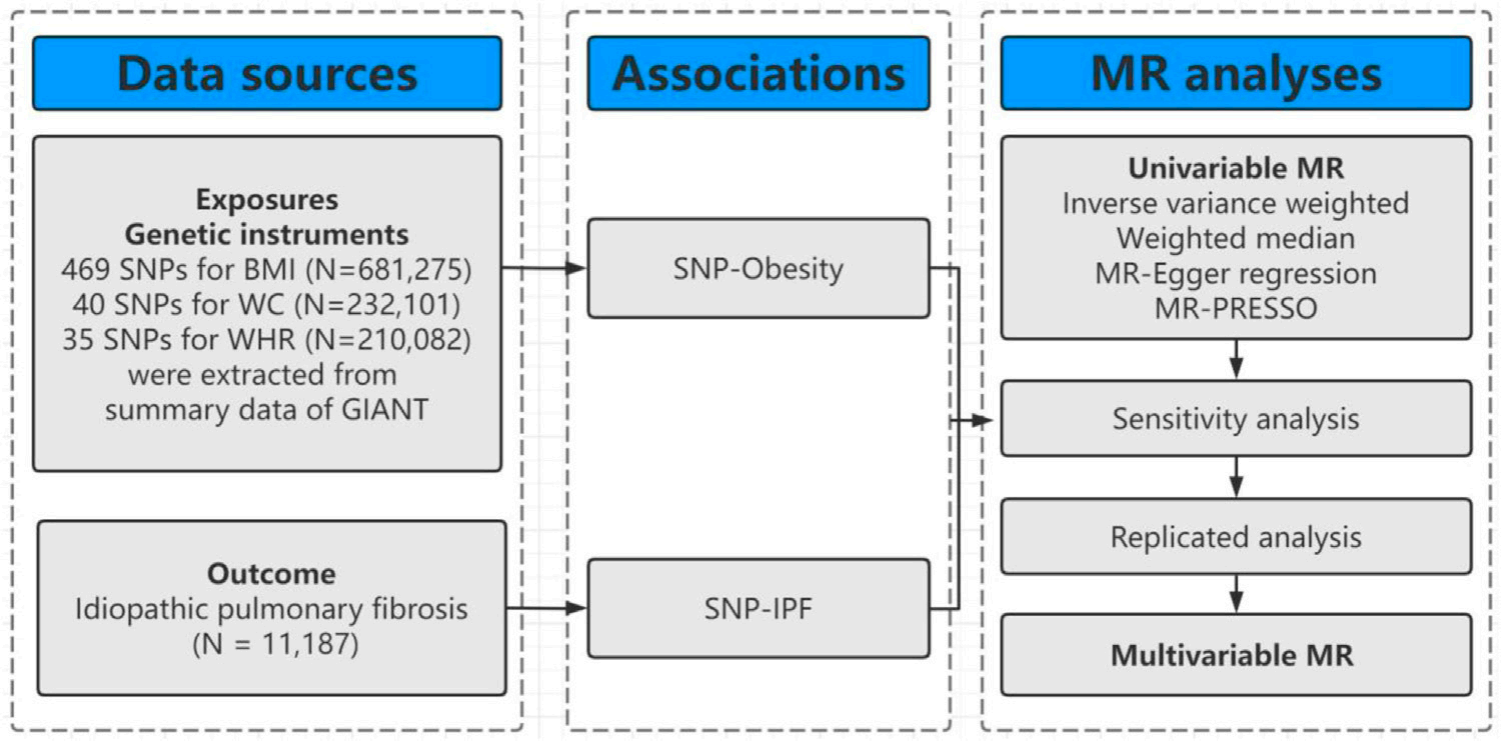
Diagram of Mendelian randomization (MR) framework in this study. SNP indicates single nucleotide polymorphism; BMI, body mass index; WC, waist circumference; WHR, waist-to-hip ratio; IPF, idiopathic pulmonary fibrosis; GIANT, Genetic Investigation of ANthropometric Traits; MR-PRESSO, MR–Pleiotropy Residual Sum and Outlier.

Figure 1 shows the design of this study. Firstly, we selected genetic variants as instrumental variables (IVs) for BMI, WC, and WHR. Secondly, we collected the complete summary data from large-scale genome-wide association studies (GWASs) for IPF. Thirdly, we performed univariate two-sample MR with three MR methods [e.g., inverse-variance weighted (IVW), MR-Egger regression, weighted median, and MR–Pleiotropy Residual Sum and Outlier (MR-PRESSO)]. Finally, we conducted a series of sensitivity analyses and multivariable MR as a validation procedure for the findings in univariable MR.

### 2.2 Genetic Variants Selection

**FIGURE 2:**
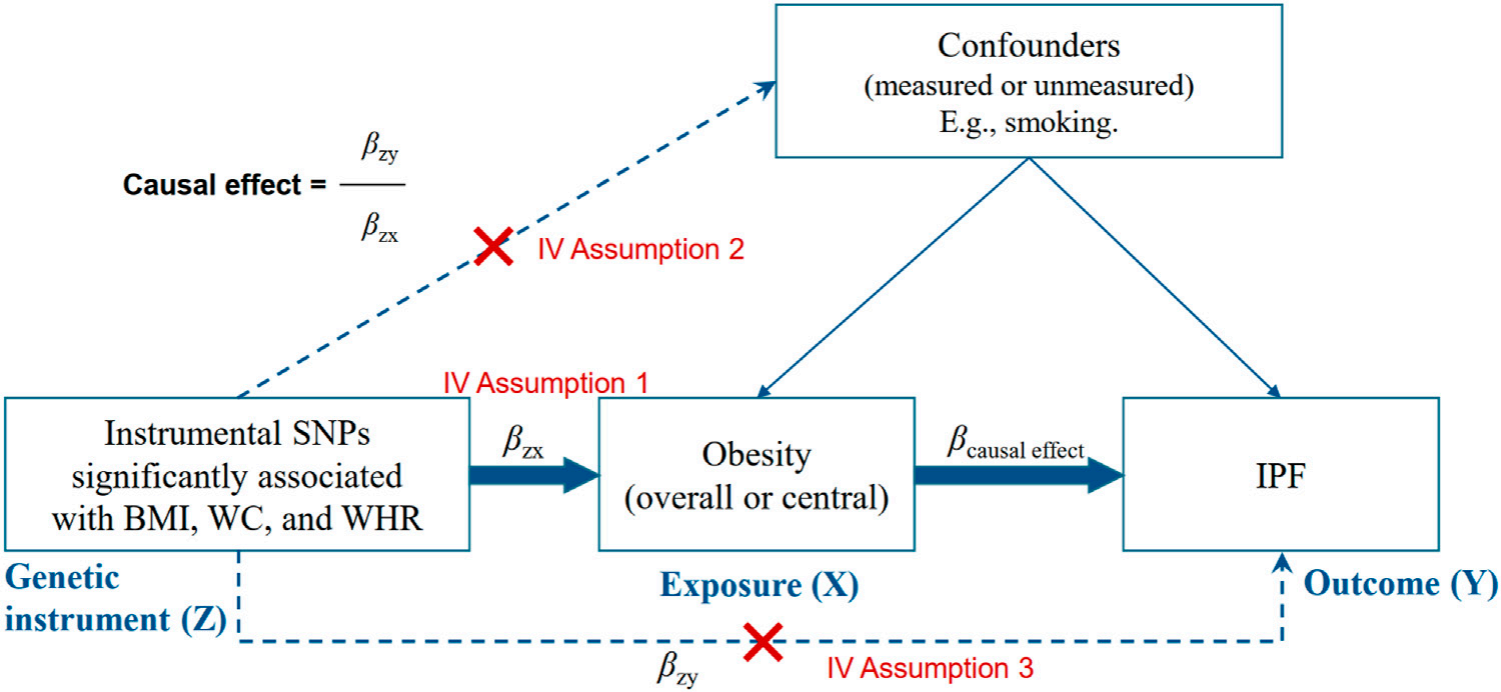
Instrumental variable (IV) assumptions of Mendelian randomization. BMI, indicates body mass index; WC, waist circumference; WHR, waist-to-hip ratio; SNP, single. nucleotide polymorphism; IPF, idiopathic pulmonary fibrosis.

We collected summary data of obesity-related traits (BMI, WC, and WHR) from the Genetic Investigation of ANthropometric Traits (GIANT) consortium (http://portals.broadinstitute.org/collaboration/giant/index.php/GIANT_consortium_data_files) and the MRC Integrative Epidemiology Unit (MRC-IEU) using the United Kingdom Biobank genetic data (https://www.mrbase.org/) (Hemani et al., 2018). The GIANT consortium has access to anthropometric and genotyping data for nearly 250,000 individuals. The United Kingdom Biobank recruited more than 500,000 individuals aged 37–73 years across the United Kingdom, between 2006 and 2010. It aimed to identify the phenotypic and health-related information by following up with participants over time. We selected obesity-related single nucleotide polymorphisms (SNPs) as IVs using a threshold *p*-value <5 × 10^–8^ (IV assumption 1, Figure 2). To minimize the influence of linkage disequilibrium (LD), which may bias the results of randomized allele allocation, a stringent condition (LD threshold of r^2^ < 0.001 and distance located 10,000 kb apart from each other) was set to ensure that the genetic instruments selected for obesity are conditionally independent to each other. The *F* statistic represents the strength of the relationship between IVs and VAT. Generally, *F* > 10 may attenuate bias of using weak genetic instruments (Burgess and Thompson, 2011). Finally, we obtained 469 SNPs for BMI (Yengo et al., 2018), 40 SNPs for WC (Shungin et al., 2015), and 35 SNPs for WHR (adjusted for BMI) (Shungin et al., 2015) as IVs to perform MR analyses.

### 2.3 Data for Outcome

We collected the summary data of IPF from the largest available GWAS to date, which was a meta-analysis of three studies including 2,668 IPF cases and 8,519 controls (Allen et al., 2020). Of these, the discovery analysis included three studies from the United Kingdom (Allen et al., 2017), Chicago (Noth et al., 2013), and Colorado (Fingerlin et al., 2013). Replication analysis was performed in two independent data sets, one from the United States, the United Kingdom, and Spain and the other from Genentech studies. All the participants were of European ancestry, as a consistent selection in exposure data.

### 2.4 Statistical Analysis

#### 2.4.1 Two-Sample Mendelian Randomization

As shown in Figure 2, we calculated the association between obesity-related traits and IPF using the basic model: β_causal effect_ = β_ZY_/β_ZX_ (β_ZY_ and β_ZX_ represent the regression coefficient on obesity-related traits and IPF, respectively). Generally, a valid IV should satisfy three assumptions (Figure 2): must be truly associated with obesity-related traits (*p* < 5 × 10^–8^) (John et al., 2020); not associated with confounders of obesity or IPF (Raghu et al., 2014); and should only be associated to the IPF through the obesity-related traits (Ley et al., 2011).

To evaluate the causal effects of obesity-related traits on the risk of IPF, we conducted a two-sample MR analysis (Burgess et al., 2015) using three MR methods, including IVW (Johnson and Uk, 2012), MR-Egger regression (Bowden et al., 2015), weighted median (Bowden et al., 2016), and MR-PRESSO (Verbanck et al., 2018). The IVW is a conventional method to obtain an MR estimate performing a meta-analysis of each Wald ratio for multiple SNP. The IVW could provide the strongest statistical power when none of the assumptions are violated. The MR-Egger regression, with the criterion relaxed, allowing horizontal pleiotropy across SNPs, also requires the Instrument Strength Independent of Direct Effect assumption. However, it provides less precise estimates. The weighted median estimator provides the median effect of SNPs, allowing up to 50% of invalid SNPs. The MR-PRESSO regresses the SNP–outcome estimates against the SNP–exposure estimates to identify outlier SNPs and outputs a corrected MR estimate.

Moreover, we used summary data from two additional studies to extract IVs for BMI (Locke et al., 2015) and WC (MRC-IEU, GWAS pipeline using PHEnome Scan ANalysis Tool–derived variables in the United Kingdom Biobank). Then, we performed a replicated MR analysis to validate the findings in the primary analysis further.

#### 2.4.2 Sensitivity Analyses for Two-Sample Mendelian Randomization

**FIGURE 3:**
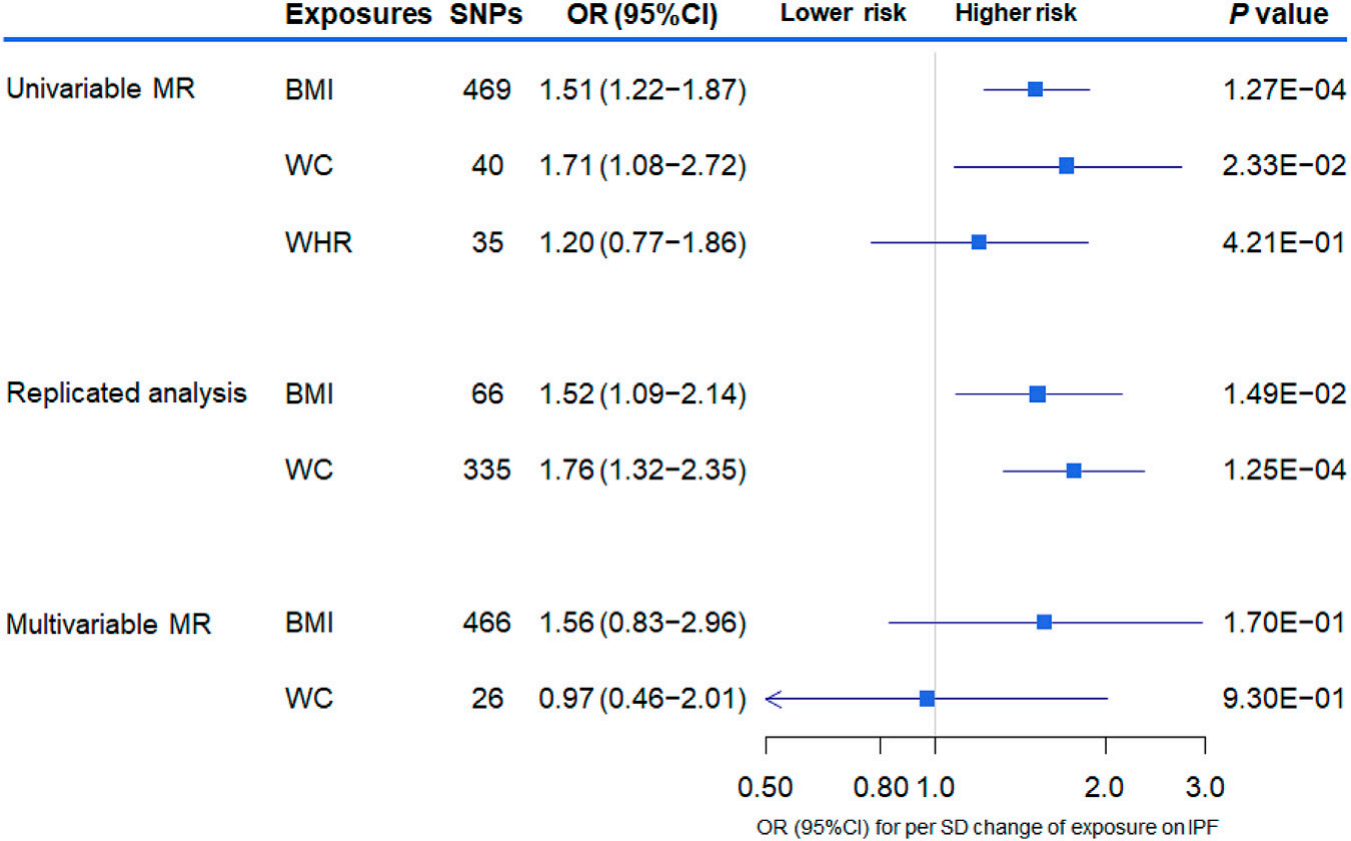
The association between genetically increased obesity-related traits and the risk of idiopathic pulmonary fibrosis (IPF) using univariable and multivariable inverse-variance weighted Mendelian randomization (MR). OR indicates odds ratio; CI, confidence interval; SNP, single nucleotide polymorphism; BMI, body mass index; WC, waist circumference; WHR, waist-to-hip ratio.

We evaluated the heterogeneity of the MR results using the Cochran's Q-test (Burgess et al., 2017) and examined the horizontal pleiotropy by testing whether the intercept in MR-Egger regression differs from zero. We also performed the leave-one-out analysis by eliminating SNPs one by one and recomputing the effect (Figure 3).

#### 2.4.3 Multivariable Mendelian Randomization to Assess the Direct Causal Effect

MR analysis adjusted for potential confounders will contribute to specifying the independent effect of obesity-related traits on IPF risk. The BMI and WC are closely related. Considering this, we also used multivariable MR (MVMR) analysis to estimate the relatively direct causal effect of BMI and WC on the risk of IPF.

Based on the above analyses, we took IVW results as the primary causal effect estimates and considered the consistency across other MR methods and MVMR. Taken together, we could conclusively establish a robustly causal relationship when satisfying the following conditions: one of the IVW and MVMR results reached the Bonferroni multiple comparison test *p*-value < 0.017 (0.05/3) (John et al., 2020); IVW and MVMR showed a similar magnitude and the same direction of effect and overlapped confidence intervals (CIs) (Raghu et al., 2014); the other MR methods showed the same direction and effect with IVW and MVMR (Ley et al., 2011).

MR analysis was performed in R (version 4.0.3) with R packages “vroom,” “tidyr,” “tibble,” “dplyr,” “TwoSampleMR” (Hemani et al., 2018), “MR-PRESSO” (Verbanck et al., 2018), and “MVMR” (Sanderson et al., 2018). The *p*-values were two-sided, and the statistical significance was set at the adjusted *p*-value < 0.017.

## 3 Results

### 3.1 Participant Characteristics and Instruments

The characteristics of the participants from the GIANT consortia for obesity-related traits and meta-analysis for IPF are shown in Table 1. A total of 469 SNPs were obtained for the BMI, 40 SNPs for WC, and 35 SNPs for WHR (adjusted for BMI), with *F* statistics ranging from 35.62 to 81.87, reflecting a strong instrument strength for obesity. A total of 66 SNPs for BMI and 335 SNPs were selected for WC extracted from the other independent GWAS summary data for further replicated analysis, with *F* statistics of 344.85 and 50.94, respectively.

**TABLE 1:** Characteristics of obesity-related consortium and idiopathic pulmonary fibrosis (IPF) data sets.

Exposures	Consortium	SNPs	*F*	Cases/controls	Sample size	Population
**Primary analysis**						
BMI	GIANT	469	81.87	NA	681,275	European
Waist circumference	GIANT	40	37.12	NA	232,101	European
Waist-to-hip ratio (BMI adjusted)	GIANT	35	35.62	NA	210,082	European
**Replicated analysis**						
Body mass index	GIANT	66	344.85	NA	322,154	European
Waist circumference	MRC-IEU	335	50.94	NA	462,166	European
**Outcome**	**Data source**	**Studies**	**—**	**Cases/controls**	**Sample size**	**Population**
IPF	Meta-analysis	3	—	2,668/8,519	11,187	European

SNP indicates single nucleotide polymorphism; *F*, *F* statistic; BMI, body mass index; GIANT, Genetic Investigation of ANthropometric Traits; MRC-IEU, MRC Integrative epidemiology unit; NA, not applicable.

### 3.2 Main Results of Univariable Two-Sample Mendelian Randomization

The overall results of the univariate MR analysis for genetically determined obesity on IPF risk are shown in Table 2. In the primary analysis, evidence showed that the per SD increase in obesity-related traits was associated with a higher risk for IPF. Specifically, the odds ratio (OR) for developing IPF per SD increase in BMI and WC was 1.51 [95% CI (1.22–1.87), *p* = 1.27 × 10^–4^] and 1.71 [95% CI (1.08–2.72), *p* = 2.33 × 10^–2^], respectively. However, there is little evidence to support an association between WHR (adjusted for BMI) and IPF. We obtained similar results in the replicated analysis, suggesting that the increased BMI and WC are associated with IPF risk [odds ratio (OR) = 1.52, 95% CI (1.09–2.14), *p* = 1.49 × 10^–2^ and OR = 1.76, 95% CI (1.32–2.35), *p* = 1.25 × 10^–4^, respectively]. Of these, the MR method of weighted median showed good consistency with IVW (*p* < 0.05) for the BMI. Subsequently, only the results for BMI and IPF survived following the multiple testing correction (adjusted *p*-value < 0.017 in both IVW and weighted median).

**TABLE 2:** Two-sample Mendelian randomization (MR) estimations showing the effect of obesity on the risk of idiopathic pulmonary fibrosis.

Methods	Exposures	Odds ratio (95% CI)	*p*-value	Power	Q-statistics	*P*_h_	Egger intercept	*P*_intercept_
MR-Egger	BMI	1.61 (0.92–2.82)	9.34E-02	1.00	481.73	3.09e-01	−0.001 (−0.010–0.008)	8.08e-01
Inverse-variance weighted		1.51 (1.22–1.87)	1.27E-04	1.00	481.79	3.20e-01		
Weighted median		1.57 (1.09–2.26)	1.43E-02	1.00	—	—		
MR-Egger	WC	2.32 (0.45–11.90)	3.20E-01	1.00	43.06	2.28e-01	−0.009 (−0.053–0.036)	7.06e-01
Inverse-variance weighted		1.71 (1.08–2.72)	2.33E-02	0.87	43.23	2.58e-01		
Weighted median		1.76 (0.91–3.43)	9.38E-02	0.91	—	—		
MR-Egger	WHR	0.71 (0.09–5.84)	7.52E-01	0.68	34.70	3.87e-01	0.015 (−0.043–0.063)	6.21e-01
Inverse-variance weighted		1.20 (0.77–1.86)	4.21E-01	0.31	34.96	4.22e-01		
Weighted median		0.85 (0.45–1.62)	6.21E-01	0.22	—	—		

CI indicates confidence interval; NA, not applicable; *P*_h_, *p*-value for heterogeneity; *P*_intercept_, *p*-value for intercept of MR-Egger regression. Power was calculated by an online tool (https://shiny.cnsgenomics.com/mRnd/). MR–Pleiotropy Residual Sum and Outlier did not identify any outliers, so the results are not shown in Table 2.

### 3.3 Sensitivity Analyses for Univariate Two-Sample Mendelian Randomization

**FIGURE 4:**
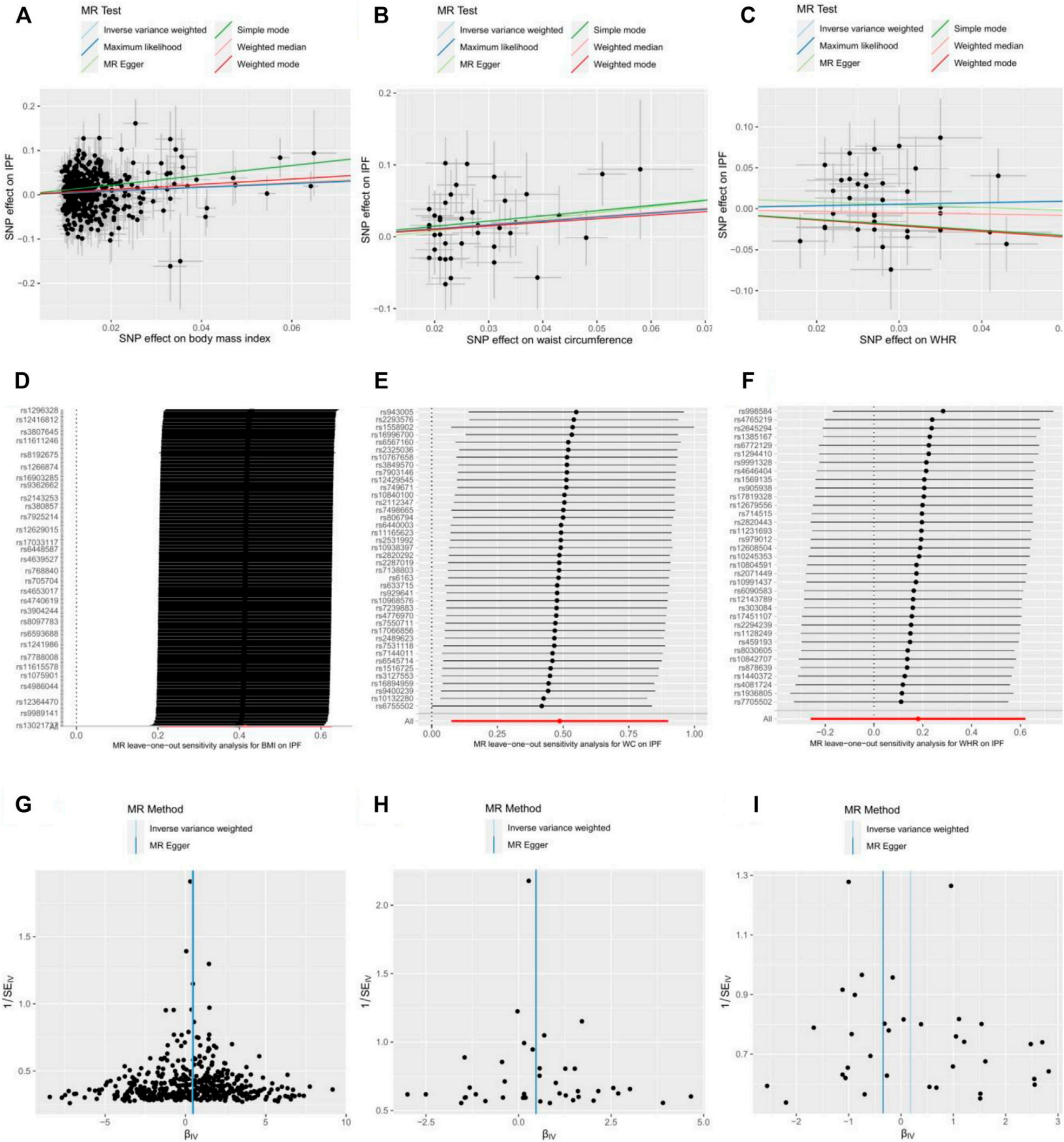
Scatter plot, leave-one-out test, and funnel plot for effects of body mass index (BMI) **(A,D,G)**; waist circumference (WC) **(B,E,H)**; and waist-to-hip ratio (WHR) **(C,F,I)** on the risk of idiopathic pulmonary fibrosis (IPF). MR indicates Mendelian randomization; SNP, single nucleotide polymorphism.

We performed a series of sensitivity analyses to evaluate the heterogeneity and potential horizontal pleiotropy (Table 2). Cochran's Q-test showed little evidence (*p*_heterogeneity_ > 0.05) for the presence of heterogeneity for the MR estimations of BMI, WC, and WHR. Additionally, the MR-Egger intercept test showed no pleiotropy (*p*_intercept_ > 0.05) for these three obesity-related traits. In the leave-one-out test, we found that the MR estimates were stable when dropping a single SNP out, one by one in BMI and WC (Figure 4). Except for WHR, the funnel plots for BMI and WC did not show any noticeable asymmetry, indicating that the MR results for BMI and WC were not influenced by horizontal pleiotropy (Figure 4).

### 3.4 Main Results of Multivariable Mendelian Randomization

We further performed multivariable MR including BMI and WC in the same model to estimate the independent effect of these two obesity-related traits on the risk of IPF. Despite the loss of statistical significance, we found that the effective magnitude and direction of increased BMI on the risk of IPF remained consistent with those of the univariable MR analysis, also sharing overlapped CIs [OR = 1.56, 95% CI (0.83–2.96), *p* = 1.70 × 10^–1^]. The magnitude and direction of MR estimate for WC on IPF risk in the univariable MR changed obviously after multivariate adjustment [OR = 0.97, 95% CI (0.46–2.01), *p* = 9.30 × 10^–1^] (Figure 3).

## 4 Discussion

In this study, we performed MR analyses to test if human genetic evidence supported a causal effect of increased obesity-related factors (BMI, WC, and WHR) on the risk of IPF. Overall, the increased BMI, other than WC or WHR, showed causal effects on the risk of IPF. Our findings were further supported by replicated analysis and multivariable MR. Combined with a series of sensitivity analyses, the association between BMI and IPF risk can therefore be considered most reliable.

The pathogenesis of IPF is complex, and the etiology remains obscure. Previous studies have focused on the nongenetic risk factors, including male sex, old age, and smoking (Baumgartner et al., 2000), or genetic evidence including several mutations identified in patients with familial pulmonary fibrosis, such as mutations in surfactant proteins (SP-A2 and SFTPC) (Wang et al., 2009; van Moorsel et al., 2010), gel-forming mucin (MUC5B) (Seibold et al., 2011), and telomerase (Armanios et al., 2007; Fukuhara et al., 2013; Duckworth et al., 2021). Some of these have also been identified in individuals with sporadic IPF, suggesting a possible genetic predisposition to IPF. However, a few observational or basic studies have specifically investigated the association between obesity and IPF. One of the reasons could be the rapid progression for IPF, leading to a marked weight loss when diagnosed, resulting in overlooking the potential effects of obesity on incident IPF. More than that, Jouneau et al. (2020) found that lower BMI and weight loss in patients with IPF may be associated with a faster decline in forced vital capacity. Notably, this study investigated the association between BMI and risk in patients who had already developed IPF, but we focused on the impact of decreased BMI on IPF prevention. In other words, compared with observational studies, MR analyses use genetic IVs to estimate the effect of exposure on disease incidence, rather than prognosis. Therefore, current observational studies and MR analyses, focusing on different perspectives, are not mutually exclusive. In addition, weight loss may be beneficial to susceptible populations for IPF perioperative treatment. For example, it has been reported that obese patients (BMI >30 kg/m^2^) with IPF who received a bilateral lung transplant may be at a higher risk of a 90-day mortality compared with patients of normal weight (Gries et al., 2015).

Inflammation may act as an intermediate link between obesity and IPF. Obesity causes a chronic systemic inflammatory state (Lumeng and Saltiel, 2011; González-Muniesa et al., 2015; Stepanikova et al., 2017), which has been linked to IPF (Desai et al., 2018; Cho et al., 2019). Inflammation preceded fibrosis in animal models with IPF, and suppression of the inflammation contributed to attenuate further fibrotic response (Yan et al., 2018). Additionally, asymptomatic relatives of patients with a familial form of IPF have cellular evidence of alveolitis before being clinically recognized (Bitterman et al., 1986). The alveolar macrophage was proposed to play a key role in the genesis of IPF due to its ability to secrete proinflammatory and profibrotic cytokines that affect mesenchymal cell proliferation and promote collagen deposition.

Early theories declaimed that recurrent damage to the alveolar epithelial cells promotes fibrogenesis in the pathogenesis of lung fibrosis (Selman and Pardo, 2006). Continuous alveolar epithelial stem cells injury or dysfunction appears to be the major initial driver of IPF (Selman and Pardo, 2020). Under the long-term stimulation of chronic inflammation, alveolar epithelial cells exhibited enhanced apoptosis and a greater ability of epithelial–mesenchymal transition (Cabrera-Benítez et al., 2012), while alveolar epithelial cells also secreted cytokines and growth factors which in turn promote apoptosis and epithelial–mesenchymal transition (Xu et al., 2016). Thus, in the presence of both inflammation and alveolar epithelial cell dysfunction, it may be more likely to develop pulmonary fibrosis.

Another characteristic feature of IPF is the progressive fibrotic process. It is now considered that the interaction of growth factors and cytokines with cells resident in the lungs is important to the fibrotic process in IPF (Wynn, 2011; Grimminger et al., 2015). Patients with IPF have a higher level of transforming growth factor-β (TGF-β) in bronchoalveolar lavage than normal controls. TGF-β is one of the most strong regulators of connective tissue synthesis and is significantly increased in patients with obesity (Woo et al., 2021). Moreover, several other obesity-related inflammatory factors, including IL-17, leptin, adiponectin, NLRP3 inflammasome, and TLR-4 have been implicated in the pathogenesis of lung disease. These mediators are known to be modulated by autophagy activity (Pabon et al., 2016). Of these, IL-17A has been shown to play an important role in pulmonary fibrosis by promoting collagen production (Samokhin et al., 2010). The regulation of IL-17 by autophagy could be altered in obesity and promote the pathogenesis of pulmonary diseases such as IPF.

Interestingly, only BMI showed a causal effect on the risk of IPF in our MR analysis. Generally, BMI represents the overall adiposity, whereas WC and WHR are markers of central obesity. This distinction could explain the significant difference in IPF incidence between Europe and North America (2.8–18 cases per 100,000 people per year) and Asia and South America (0.5–4.2 cases per 100,000 people per year) (Hutchinson et al., 2015; Hopkins et al., 2016; Richeldi et al., 2017).

To the best of our knowledge, this study is the first in estimating obesity on IPF risk using MR methods. We applied a series of sensitivity analyses, as well as multivariable MR to minimize the influence of potential confounders and horizontal pleiotropy. There are also three major limitations in our study: we could not make a gender-stratification MR analysis for IPF because the author did not provide gender-specific data (John et al., 2020); only Europeans were included in our MR analysis, limiting the generalization of the conclusions to other ethnicities (Raghu et al., 2014); there was a risk of false-positive in this study, which may be caused by the overlapping samples between exposure and outcome data (Ley et al., 2011). After calculation, however, we obtained a maximum overlapping rate of 0.49%, which may not have been sufficient to affect our results.

## 5 Conclusion

In summary, our MR results provided evidence that genetically determined BMI increment demonstrates a higher risk on IPF, expanding our knowledge of additionally novel etiology for IPF. Further studies are required to determine the reliability of BMI as a predictor of IPF risk, to evaluate the mediating mechanisms for potential intervention targets.

## Data Availability

The original contributions presented in the study are included in the article/Supplementary Material, and further inquiries can be directed to the corresponding authors.
