# Supplementary material for: Management of BMI Is a Potential New Approach for the Prevention of Idiopathic Pulmonary Fibrosis
Source: Front Genet. 2022 Mar 11;13:821029. doi: 10.3389/fgene.2022.821029 (PMC8961741; doi:10.3389/fgene.2022.821029)
Supplement: Supplementary file 1 [file DataSheet1.ZIP › Supplementary Material Presentation Figure 1-4/Figure 1-4.pdf]

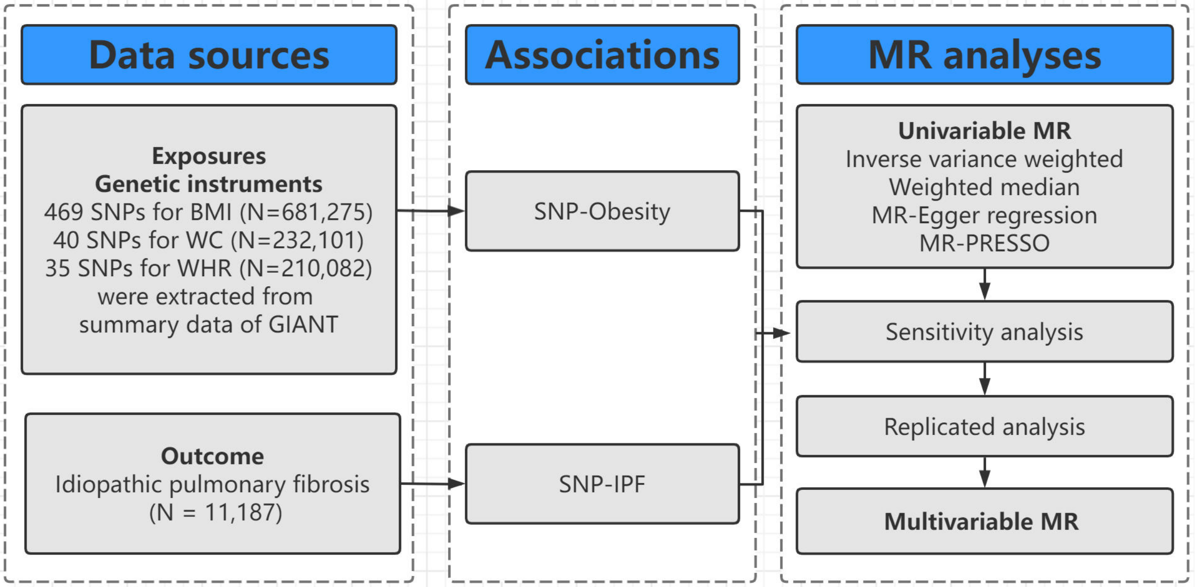

Figure 1. Diagram of Mendelian randomization framework in this study.

SNP indicates single nucleotide polymorphism; BMI, body mass index; WC, waist circumference; WHR, waist-to-hip ratio; MR, Mendelian randomization; IPF, idiopathic pulmonary fibrosis.

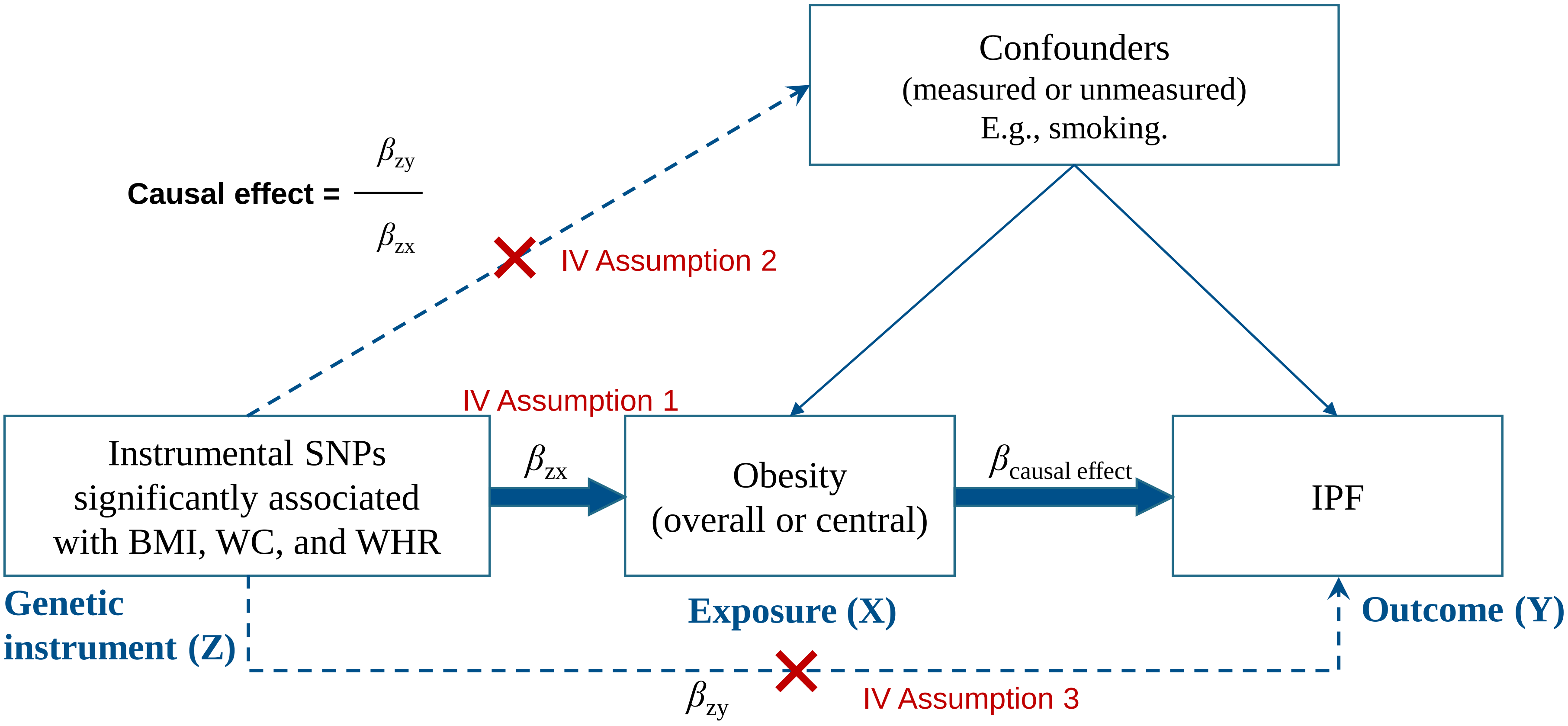

Figure 2. Instrumental variable (IV) assumptions of Mendelian randomization.

BMI, indicates body mass index; WC, waist circumference; WHR, waist-to-hip ratio; SNP, single nucleotide polymorphism; IPF, idiopathic pulmonary fibrosis.

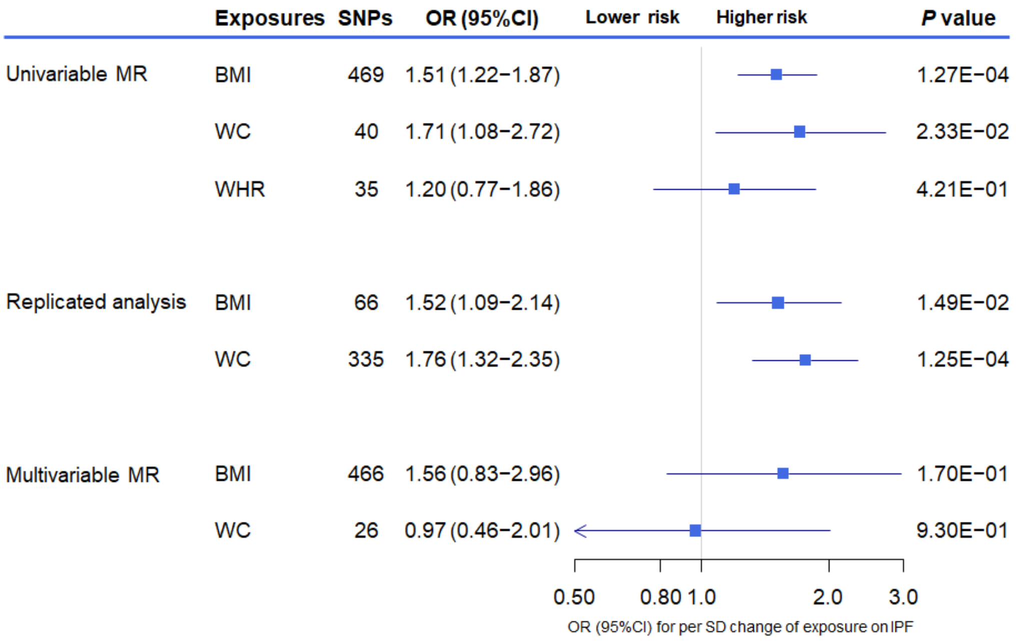

Figure 3. The association between genetically increased obesity-related traits and the risk of idiopathic pulmonary fibrosis using univariable and multivariable IVW MR.

OR indicates odds ratio; SNP indicates single nucleotide polymorphism; BMI, body mass index; WC, waist circumference; WHR, waist-to-hip ratio; MR, Mendelian randomization; IPF, idiopathic pulmonary fibrosis.

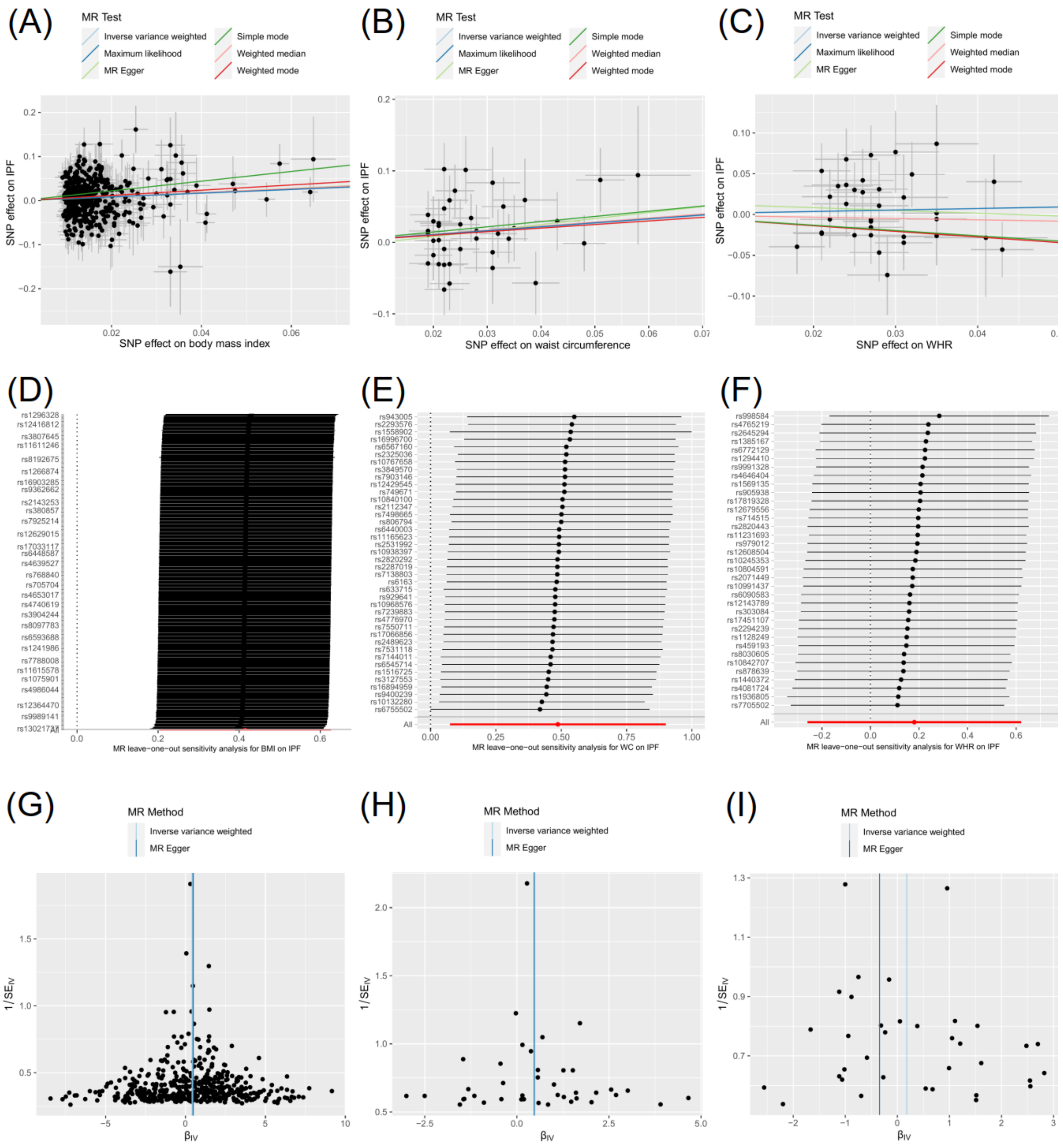

Figure 4. Scatter plot, leave-one-out test, and funnel plot for effects of BMI (A, D, and G), WC (B, E, and H), and WHR (C, F, and I) on the risk of idiopathic pulmonary fibrosis.

BMI indicates body mass index; WC, waist circumference; WHR, waist-to-hip ratio; MR, Mendelian randomization; IPF, idiopathic pulmonary fibrosis.
