# Supplementary material for: Management of BMI Is a Potential New Approach for the Prevention of Idiopathic Pulmonary Fibrosis
Source: Front Genet. 2022 Mar 11;13:821029. doi: 10.3389/fgene.2022.821029 (PMC8961741; doi:10.3389/fgene.2022.821029)
Supplement: Supplementary file 2 [file DataSheet2.ZIP › Supplementary Material Presentation Table S1/Table S1.pdf]

Table S1. Instrumental variables for body mass index, waist circumference, and waist-to-hip ratio.

SNP	EA	OA	beta	beta	eaf	chr	pos	se	pval	samplesize	exposure
rs10009336	T	C	-0.014	-0.0336	0.1638	4	44480783	0.0022	2.20E-10	794766	BMI
rs10132280	A	C	-0.022	-0.10245	0.3017	14	25928179	0.0018	5.60E-35	786578	BMI
rs10169594	C	T	0.0121	0.022379	0.3596	2	41637688	0.0018	2.00E-11	685712	BMI
rs10182181	G	A	0.0325	0.048488	0.4753	2	25150296	0.0016	6.70E-90	792111	BMI
rs10192119	G	T	0.0166	0.04646	0.1673	2	1.65E+08	0.0022	3.00E-14	795369	BMI
rs10197031	C	T	0.0166	-0.00981	0.2834	2	1.05E+08	0.0019	1.90E-18	691479	BMI
rs10247983	A	G	0.0201	-0.08886	0.9213	7	1.15E+08	0.0033	1.70E-09	672411	BMI
rs10248136	T	C	-0.01	-0.00693	0.5142	7	39077397	0.0017	2.00E-08	686892	BMI
rs10269783	A	G	0.0133	0.032125	0.3896	7	49616203	0.0017	1.40E-15	790551	BMI
rs10408324	T	C	-0.012	-0.0469	0.2744	19	51774806	0.0019	9.50E-11	690737	BMI
rs10478110	C	A	0.01	-0.04397	0.4348	5	1.12E+08	0.0017	9.60E-09	680441	BMI
rs1048932	A	C	-0.016	0.046157	0.4162	11	1.15E+08	0.0017	3.80E-22	795167	BMI
rs10492229	T	C	0.0142	-0.04831	0.2268	12	1.11E+08	0.0019	7.70E-14	794845	BMI
rs10510419	T	G	-0.018	-0.05101	0.1416	3	12426936	0.0023	2.20E-14	789318	BMI
rs10518694	A	C	0.0146	0.016751	0.1424	15	53072673	0.0025	3.30E-09	690554	BMI
rs1064213	A	G	0.012	0.006426	0.492	2	1.99E+08	0.0017	2.40E-12	692576	BMI
rs10733051	G	A	-0.01	-0.00436	0.4802	1	1.67E+08	0.0016	2.90E-09	781928	BMI
rs10742752	C	T	0.0124	0.040851	0.6159	11	45438374	0.0017	1.10E-13	792704	BMI
rs10747488	A	C	-0.012	-0.06219	0.7601	1	98299475	0.002	1.20E-09	689295	BMI
rs10750215	T	G	0.0108	-0.00081	0.3883	11	1.23E+08	0.0017	1.30E-10	788895	BMI
rs1075901	C	T	0.0121	0.041231	0.5639	17	15943910	0.0016	1.20E-13	794789	BMI
rs10768994	C	T	-0.011	0.033794	0.4337	11	43936945	0.0017	6.40E-12	791685	BMI
rs10795422	G	A	0.0139	0.028317	0.6905	10	16759312	0.0019	9.30E-14	692108	BMI
rs10811871	G	A	-0.011	-0.06718	0.3829	9	23200766	0.0018	1.60E-09	686376	BMI
rs10832778	G	C	0.0125	-0.00442	0.6222	11	17394073	0.0017	1.30E-13	783042	BMI
rs10858334	G	C	0.0143	-0.03396	0.1415	9	1.38E+08	0.0026	2.70E-08	672640	BMI
rs10867256	T	C	-0.012	-0.01085	0.553	9	81367391	0.0017	8.70E-12	689493	BMI
rs10878946	T	C	-0.014	0.047028	0.714	12	69642315	0.0019	3.60E-13	685707	BMI
rs10914462	G	A	-0.011	-0.02065	0.4255	1	32125943	0.0017	1.50E-10	689808	BMI
rs10915840	A	G	-0.012	0.013574	0.283	1	2.26E+08	0.0019	1.30E-09	684857	BMI
rs10920678	G	A	-0.016	-9.56E-05	0.5709	1	1.9E+08	0.0016	1.50E-21	788624	BMI
rs10938397	G	A	0.0324	0.01222	0.4317	4	45182527	0.0016	3.40E-86	793518	BMI
rs10942267	G	A	-0.016	-0.00715	0.3088	5	80841914	0.0019	3.90E-17	689084	BMI
rs10953740	G	A	-0.015	-0.00079	0.5534	7	1.13E+08	0.0017	1.00E-18	684419	BMI
rs10962550	C	G	0.0182	0.010756	0.1801	9	16720329	0.0022	6.20E-16	690579	BMI
rs10971709	T	C	0.0132	0.023173	0.2062	9	33804813	0.0021	6.20E-10	688312	BMI

rs10984756	G	C	0.0174	0.128167	0.1048	9	1.23E+08	0.0029	1.10E-09	689917	BMI
rs11030618	T	C	0.011	-0.01181	0.5679	11	29243293	0.0017	2.40E-10	690005	BMI
rs11066188	A	G	-0.012	0.067525	0.4181	12	1.13E+08	0.0017	8.10E-13	792755	BMI
rs11084553	G	A	-0.021	-0.02558	0.1518	19	31019780	0.0024	1.80E-18	691103	BMI
rs11105839	A	T	-0.011	-0.01904	0.3799	12	91237920	0.0017	1.10E-10	781573	BMI
rs11115176	C	T	-0.012	-0.00334	0.2399	12	82465797	0.0019	2.00E-10	792384	BMI
rs11118308	G	A	-0.01	-0.01935	0.4703	1	2.2E+08	0.0016	4.80E-10	794625	BMI
rs1112613	A	G	-0.013	0.003758	0.1762	13	53651850	0.0023	3.40E-09	682816	BMI
rs11150911	C	A	-0.013	0.079585	0.7191	18	73498528	0.0018	4.70E-13	781716	BMI
rs11165643	T	C	0.0206	-0.02687	0.5828	1	96924097	0.0017	1.40E-35	792657	BMI
rs11170468	C	A	-0.012	-0.02956	0.2326	12	39430048	0.0019	1.90E-10	795265	BMI
rs11173522	A	C	0.0128	-0.04617	0.2078	12	60953472	0.0021	1.10E-09	691593	BMI
rs11185111	A	G	-0.013	0.024571	0.3042	1	1.08E+08	0.0019	7.70E-12	686508	BMI
rs11251352	G	A	0.0109	-0.00551	0.5988	10	2585792	0.0018	7.00E-10	690804	BMI
rs11496125	T	C	0.0169	-0.01089	0.4212	7	1.03E+08	0.0017	3.00E-22	684574	BMI
rs11505821	T	A	0.0311	0.071165	0.0601	7	76818677	0.0035	2.70E-19	758322	BMI
rs11538	G	A	0.0135	0.006238	0.1805	22	18220831	0.0023	3.30E-09	692349	BMI
rs1158805	A	C	-0.014	-0.03062	0.3766	18	40736590	0.0018	1.20E-14	691776	BMI
rs11609659	C	T	-0.015	-0.04195	0.2371	12	1.08E+08	0.002	2.20E-14	679177	BMI
rs11611246	T	G	0.024	-0.02732	0.21	12	939480	0.002	5.00E-32	779823	BMI
rs11615578	T	C	0.013	0.044376	0.2474	12	1.22E+08	0.002	8.10E-11	669422	BMI
rs11656076	A	G	-0.014	-0.02646	0.2254	17	31464270	0.0021	5.60E-12	691283	BMI
rs11672660	T	C	-0.034	-0.02432	0.2049	19	46180184	0.0021	1.70E-60	768426	BMI
rs11713193	A	G	0.0239	-0.00092	0.5073	3	49924424	0.0017	2.40E-44	692159	BMI
rs11736228	T	A	-0.014	-0.09305	0.2587	4	1.47E+08	0.002	4.10E-12	691580	BMI
rs11738695	A	C	0.0097	0.032125	0.586	5	1.09E+08	0.0017	2.00E-08	691380	BMI
rs11739877	T	C	0.0117	0.03099	0.6118	5	1.06E+08	0.0018	6.60E-11	692540	BMI
rs11781699	C	T	0.0132	-0.00319	0.1896	8	1.19E+08	0.0021	3.10E-10	784642	BMI
rs1187352	C	T	0.0119	0.06207	0.6518	9	87293457	0.0018	6.00E-11	688522	BMI
rs11880870	G	A	-0.019	-0.0175	0.4801	19	18830704	0.0017	1.00E-28	717350	BMI
rs11889536	G	A	-0.019	0.024605	0.1493	2	2.2E+08	0.0024	6.40E-15	688977	BMI
rs11908637	A	G	-0.012	0.032796	0.236	20	47428485	0.0021	4.90E-09	691443	BMI
rs11945861	A	G	-0.015	-0.03372	0.2369	4	65700865	0.002	5.00E-13	682451	BMI
rs11951673	T	C	-0.012	-0.06578	0.3941	5	95861012	0.0017	1.10E-13	792278	BMI
rs12033257	G	A	-0.015	-0.02606	0.3835	1	1.12E+08	0.0018	2.40E-15	664083	BMI
rs12041258	C	T	-0.015	0.044322	0.2287	1	1.95E+08	0.002	9.50E-13	688602	BMI
rs12044597	G	A	0.0143	-0.04322	0.5029	1	1708801	0.0016	1.70E-18	789125	BMI
rs12049202	T	C	0.024	0.039851	0.203	1	77967523	0.0022	1.00E-28	691566	BMI

rs12098284	T	C	0.0178	0.075978	0.1241	10	76047464	0.0026	1.80E-11	686167	BMI
rs12150665	C	T	-0.016	0.009161	0.4058	17	34914787	0.0017	1.60E-22	795501	BMI
rs1218822	A	G	0.0168	0.049002	0.6663	13	28011963	0.0017	1.90E-22	794711	BMI
rs12299814	A	C	-0.016	-0.00659	0.2525	12	90216146	0.002	5.20E-15	687962	BMI
rs12328930	C	T	0.0098	0.064272	0.4235	2	1.75E+08	0.0017	1.80E-08	690521	BMI
rs12334877	A	G	-0.014	-0.05871	0.198	8	67194171	0.0022	7.70E-11	683820	BMI
rs12364470	G	T	0.0178	0.071973	0.1626	11	1.35E+08	0.0022	1.10E-15	787411	BMI
rs12369179	T	C	-0.036	-0.06173	0.08782	12	1.23E+08	0.0031	2.50E-31	674260	BMI
rs12416812	A	G	0.0111	-0.07686	0.5088	11	888632	0.0016	6.10E-12	793338	BMI
rs1241986	A	G	-0.014	-0.04443	0.8479	18	6873954	0.0024	1.10E-08	687757	BMI
rs12422552	C	G	-0.013	0.072157	0.2663	12	14413931	0.002	1.60E-11	689543	BMI
rs12429545	A	G	0.0316	-0.03578	0.1248	13	54102206	0.0025	9.60E-38	778918	BMI
rs12448257	A	G	0.0184	0.046666	0.218	16	3599655	0.002	8.10E-20	779628	BMI
rs12564992	G	A	0.0196	0.024678	0.1144	1	1.74E+08	0.0026	5.30E-14	795119	BMI
rs12593036	G	A	-0.015	0.010946	0.2993	15	81058652	0.0019	3.80E-16	686055	BMI
rs12602912	T	C	0.0176	0.066401	0.2048	17	65870073	0.0021	9.90E-18	777510	BMI
rs1260326	C	T	0.0105	0.019854	0.5973	2	27730940	0.0017	3.90E-10	784462	BMI
rs12629015	G	A	-0.014	0.00904	0.1852	3	1.2E+08	0.0023	2.10E-09	691059	BMI
rs1266874	G	A	0.014	-0.02585	0.3558	6	51779638	0.0018	9.80E-15	691020	BMI
rs12675063	T	A	0.0156	-0.02498	0.1131	8	1.33E+08	0.0026	1.30E-09	789771	BMI
rs1268065	A	G	-0.01	0.000197	0.4794	6	1.26E+08	0.0017	1.00E-09	759626	BMI
rs12680842	G	A	-0.013	0.045277	0.3205	8	95582606	0.0018	4.40E-14	782549	BMI
rs12718572	T	C	-0.012	0.04959	0.4024	7	50573325	0.0018	3.00E-11	686523	BMI
rs12762034	C	T	0.024	-0.04709	0.07583	10	33969931	0.0032	7.30E-14	692191	BMI
rs12779328	T	C	0.0105	-0.00315	0.2833	10	12943973	0.0019	4.50E-08	690736	BMI
rs1285997	G	C	0.0142	-0.0398	0.7153	14	91513029	0.0019	1.20E-13	684235	BMI
rs12888545	G	A	0.0136	-0.09888	0.2519	14	88308044	0.002	9.10E-12	688605	BMI
rs12888955	A	G	-0.018	-0.04236	0.6513	14	1.03E+08	0.0018	1.40E-22	691849	BMI
rs12905439	G	C	-0.012	0.008396	0.3393	15	99521883	0.0018	1.40E-10	675205	BMI
rs12914489	A	G	0.0165	0.003097	0.1103	15	74187937	0.0026	3.80E-10	795244	BMI
rs12922346	C	G	0.0136	0.018609	0.2657	16	82438337	0.002	1.00E-11	679615	BMI
rs12933482	G	A	0.0186	-0.04931	0.1048	16	72189604	0.0028	4.90E-11	691477	BMI
rs12936083	G	A	0.0139	0.033908	0.3267	17	4801887	0.0019	4.10E-13	633615	BMI
rs12939549	G	A	-0.018	-0.01811	0.4335	17	78611724	0.0016	2.70E-28	793950	BMI
rs1296328	C	A	-0.018	0.067722	0.5657	4	1.37E+08	0.0018	4.90E-24	683488	BMI
rs12981256	A	G	0.0142	0.029373	0.5325	19	1865901	0.0018	1.10E-15	678327	BMI
rs13021737	G	A	0.0574	0.083828	0.8319	2	632348	0.0021	7.50E-157	789534	BMI
rs13047416	G	C	-0.015	-0.02456	0.3769	21	40309436	0.0018	2.20E-17	683228	BMI

rs13069244	A	G	0.0187	-0.03545	0.07747	3	1.8E+08	0.0032	3.00E-09	791327	BMI
rs13107325	T	C	0.047	0.037867	0.07373	4	1.03E+08	0.0032	1.10E-47	792045	BMI
rs13110266	A	G	-0.012	-0.05147	0.4065	4	1.62E+08	0.0017	1.90E-12	791087	BMI
rs13147390	C	T	0.0103	0.00211	0.3569	4	80712000	0.0018	1.00E-08	681032	BMI
rs13174863	G	A	0.0192	-0.03499	0.1548	5	1.39E+08	0.0023	2.90E-16	773762	BMI
rs13184896	T	G	-0.013	-0.00337	0.4346	5	1.23E+08	0.0016	3.30E-16	794825	BMI
rs13191362	G	A	-0.024	-0.03475	0.1198	6	1.63E+08	0.0025	5.90E-21	792699	BMI
rs1320903	A	G	0.0216	0.025822	0.3174	3	1.32E+08	0.0018	9.20E-32	691519	BMI
rs1321432	C	A	0.0201	0.009549	0.6321	20	6614691	0.0018	3.50E-29	686481	BMI
rs13240600	G	A	-0.02	0.005626	0.1552	7	99064466	0.0024	3.50E-17	692233	BMI
rs13250058	T	G	0.0112	-0.02122	0.6771	8	1.12E+08	0.0018	2.90E-10	787870	BMI
rs13263601	C	A	0.0154	0.002816	0.3478	8	14095900	0.0018	2.20E-17	686196	BMI
rs1327259	G	A	-0.016	0.051102	0.3872	6	51177811	0.0018	1.70E-18	685922	BMI
rs13287131	C	T	0.0123	0.043118	0.2491	9	92119579	0.002	6.80E-10	683808	BMI
rs1330052	G	C	0.0132	0.013731	0.3504	13	86536006	0.0018	1.50E-13	691613	BMI
rs13329567	T	C	-0.029	0.014868	0.2308	15	68104367	0.002	1.00E-50	793953	BMI
rs1365466	T	C	-0.014	0.018016	0.7406	18	36182440	0.0019	3.30E-13	791868	BMI
rs1371108	A	C	0.0119	0.042229	0.3247	2	81816251	0.0018	9.00E-11	684620	BMI
rs1409818	T	C	0.0201	0.032249	0.1156	20	21381121	0.0029	2.50E-12	690984	BMI
rs1412235	C	G	0.0246	0.022619	0.3175	9	28410996	0.0017	6.00E-45	790147	BMI
rs1421334	C	A	-0.013	-0.0794	0.5431	8	30865733	0.0018	1.00E-12	680665	BMI
rs1430387	C	T	-0.011	0.015646	0.4295	18	58227112	0.0017	5.80E-11	689325	BMI
rs1431659	G	A	-0.02	-0.02807	0.7344	8	73439070	0.0019	6.00E-24	689739	BMI
rs1436344	C	G	0.0141	0.011875	0.5922	3	1.05E+08	0.0017	4.10E-16	692017	BMI
rs1445652	A	G	0.0123	-0.02744	0.1855	2	1.56E+08	0.0022	4.30E-08	682166	BMI
rs1452075	T	C	0.0141	-0.021	0.7277	3	62481063	0.0018	1.30E-14	783729	BMI
rs1465900	C	A	-0.013	0.018629	0.2188	11	76473138	0.002	4.80E-10	779748	BMI
rs1472169	T	C	-0.014	0.009731	0.3772	9	37209396	0.0018	2.80E-15	689945	BMI
rs1477199	G	A	0.0228	-0.03217	0.1451	16	53712135	0.0024	9.40E-22	794442	BMI
rs1492767	T	C	0.0094	-0.03567	0.4957	4	55221467	0.0016	1.00E-08	794161	BMI
rs1503526	C	T	0.014	0.017906	0.4838	5	63020706	0.0017	5.50E-17	747347	BMI
rs1522569	G	T	-0.016	-0.06026	0.1819	4	1.72E+08	0.0022	2.90E-13	689573	BMI
rs1528435	T	C	0.0164	0.021501	0.6331	2	1.82E+08	0.0017	9.10E-23	794198	BMI
rs1535660	C	T	-0.015	0.030911	0.8554	9	10371073	0.0025	5.20E-09	690624	BMI
rs1538247	C	T	0.0108	0.020126	0.3181	6	1.53E+08	0.0019	1.00E-08	682726	BMI
rs156201	C	G	0.0123	0.005907	0.7606	6	1.05E+08	0.002	5.80E-10	691835	BMI
rs1624134	C	G	0.0101	0.011777	0.4068	10	34834482	0.0018	1.10E-08	690572	BMI
rs1656377	C	T	0.0099	-0.0363	0.5885	3	1.58E+08	0.0017	1.60E-08	691955	BMI

rs1681740	C	A	-0.012	-0.03137	0.3933	10	1.19E+08	0.0018	1.10E-10	673459	BMI
rs16849710	G	A	-0.012	0.034812	0.515	1	2.02E+08	0.0018	6.00E-11	666229	BMI
rs16851483	T	G	0.0369	0.019797	0.06928	3	1.41E+08	0.0035	3.20E-26	692316	BMI
rs16871902	A	G	0.0125	0.040842	0.4877	5	3488462	0.0017	4.60E-13	690830	BMI
rs16903285	C	T	0.0331	-0.00875	0.1407	5	87978252	0.0026	7.60E-38	687944	BMI
rs17001561	A	G	0.0151	-0.0034	0.1573	4	77096118	0.0023	3.80E-11	794327	BMI
rs17014375	G	T	0.0172	0.079405	0.1348	1	2.1E+08	0.0025	1.10E-11	690856	BMI
rs17033117	T	C	0.0137	-0.00357	0.1872	3	35443653	0.0022	8.90E-10	691863	BMI
rs17056301	C	T	0.0118	-0.04302	0.2636	5	1.58E+08	0.002	2.40E-09	688085	BMI
rs17113297	T	C	0.0166	0.050435	0.2082	10	1.02E+08	0.0021	2.10E-15	686357	BMI
rs17119937	C	T	0.0212	-0.0101	0.06905	8	14502274	0.0036	5.60E-09	668272	BMI
rs17203016	G	A	0.015	0.069159	0.196	2	2.08E+08	0.002	2.10E-13	786272	BMI
rs17207196	T	C	-0.022	-0.03097	0.4118	7	75101065	0.0018	2.10E-35	668894	BMI
rs17238110	G	A	-0.035	0.150008	0.1634	15	62150364	0.005	2.00E-12	775505	BMI
rs17311369	T	C	-0.01	0.018145	0.3278	15	47709199	0.0019	3.10E-08	673102	BMI
rs17399237	C	T	-0.013	-0.04877	0.5497	2	35471626	0.0017	6.70E-14	690950	BMI
rs17405819	C	T	-0.022	0.032908	0.301	8	76806584	0.0018	4.30E-33	795493	BMI
rs17424296	A	G	-0.011	0.009947	0.3659	5	60838903	0.0018	2.40E-09	684366	BMI
rs17425707	C	T	0.0167	0.072162	0.1003	1	57874879	0.0028	4.40E-09	688867	BMI
rs17446257	A	G	0.0153	0.005131	0.1292	13	40749213	0.0026	2.90E-09	690630	BMI
rs17499593	G	C	0.0125	0.052586	0.1897	2	1.73E+08	0.0022	1.10E-08	691663	BMI
rs17513613	C	T	0.0186	-0.00184	0.3236	19	30286822	0.0018	3.60E-26	789575	BMI
rs175165	G	T	-0.01	-0.01919	0.3941	22	20116015	0.0018	5.20E-09	690545	BMI
rs17535749	A	G	0.015	0.053052	0.1023	3	10027724	0.0027	2.50E-08	777038	BMI
rs17551974	A	C	-0.014	-0.03354	0.1782	2	1.42E+08	0.0022	1.90E-10	691115	BMI
rs17636031	C	T	0.016	0.01407	0.2701	10	1.27E+08	0.0019	1.20E-17	782807	BMI
rs17663412	A	C	0.0157	-0.0185	0.1139	5	1.68E+08	0.0027	6.10E-09	691018	BMI
rs17710386	C	T	0.0126	0.020486	0.3319	18	63461201	0.0018	1.00E-12	783810	BMI
rs17724992	G	A	-0.018	0.04334	0.2596	19	18454825	0.0019	1.00E-22	785851	BMI
rs17789218	C	T	0.013	0.04348	0.2392	6	1.01E+08	0.0019	7.40E-12	793904	BMI
rs17806379	T	C	-0.026	0.056568	0.1789	20	51107290	0.0022	1.50E-30	690043	BMI
rs1784460	A	T	0.0132	-0.05749	0.4035	11	1.19E+08	0.0018	9.00E-14	680042	BMI
rs1804528	A	G	0.0109	0.074053	0.3507	4	1.46E+08	0.002	3.00E-08	518856	BMI
rs1830074	C	T	0.0115	0.042511	0.288	7	6718674	0.0019	1.40E-09	689911	BMI
rs1836303	G	A	0.0116	-0.00866	0.3873	15	46539116	0.0018	5.30E-11	688991	BMI
rs1863652	A	G	-0.012	-0.00226	0.3449	4	95991417	0.0018	1.40E-10	692539	BMI
rs1884389	T	C	-0.01	0.038879	0.4289	20	1410582	0.0017	4.00E-09	683669	BMI
rs1885728	A	G	0.0108	0.044993	0.6787	6	5977833	0.0019	1.00E-08	682316	BMI

rs1891216	G	T	0.0107	-0.03779	0.3759	1	7728391	0.0018	2.40E-09	685079	BMI
rs1896767	A	G	-0.011	-0.00826	0.5376	16	62838304	0.0017	2.40E-10	686262	BMI
rs1927790	C	T	0.0148	0.021276	0.4109	13	96922191	0.0016	1.80E-19	794326	BMI
rs1928295	C	T	-0.014	-0.02189	0.4461	9	1.2E+08	0.0016	5.40E-18	793649	BMI
rs1937683	T	C	0.0109	-0.01717	0.6699	10	53679060	0.0018	3.20E-09	692539	BMI
rs1948080	G	T	-0.014	-0.02211	0.3749	9	11852043	0.0018	1.10E-14	690633	BMI
rs1982441	T	G	0.0175	-0.06108	0.1381	8	28021769	0.0026	7.00E-12	687705	BMI
rs1982725	T	C	0.0097	0.056089	0.4778	19	30618771	0.0017	3.30E-08	683155	BMI
rs1993709	G	A	0.0331	0.048884	0.8177	1	72838529	0.0021	1.90E-57	786001	BMI
rs2007231	T	C	-0.01	0.002386	0.6387	1	1.15E+08	0.0018	5.20E-09	691969	BMI
rs200810	C	T	-0.014	0.00249	0.3716	6	97922184	0.0017	5.50E-16	793699	BMI
rs2009416	T	C	-0.012	0.043542	0.361	5	92415111	0.0018	1.10E-11	691741	BMI
rs2051559	C	T	0.0176	0.055295	0.1308	4	3298800	0.0026	5.00E-12	689307	BMI
rs2065418	G	T	-0.017	0.002046	0.3623	11	30422068	0.0018	3.60E-20	691707	BMI
rs208015	C	T	-0.036	-0.08613	0.9216	17	46252346	0.0034	1.40E-25	691575	BMI
rs2124499	C	G	-0.012	-0.04035	0.3718	3	1.23E+08	0.0017	3.40E-13	785955	BMI
rs2143253	A	G	-0.019	0.022114	0.1189	20	41987392	0.0026	1.10E-12	684760	BMI
rs215634	G	A	-0.015	-0.01667	0.6212	7	32369148	0.0018	2.60E-17	681296	BMI
rs2162524	C	T	0.0155	0.04948	0.3321	2	2.31E+08	0.0018	4.10E-17	691302	BMI
rs2174307	C	G	0.0121	-0.03374	0.4067	9	73791849	0.0017	4.90E-12	686559	BMI
rs217671	G	A	0.0144	0.018476	0.2719	14	62360464	0.0019	1.30E-13	691456	BMI
rs2228213	A	G	-0.014	0.044014	0.3481	6	12124855	0.0017	4.60E-16	795595	BMI
rs2235564	T	C	0.0131	0.014741	0.3466	1	6713114	0.0018	3.70E-13	691544	BMI
rs2246012	C	T	0.0158	-0.03846	0.1628	6	1.32E+08	0.0022	3.10E-13	795598	BMI
rs2283093	T	C	0.0127	-0.0319	0.2066	7	1.27E+08	0.0021	3.10E-09	691773	BMI
rs2306537	G	A	0.0133	-0.02037	0.3092	12	1.33E+08	0.0019	8.70E-13	691923	BMI
rs2307111	C	T	-0.027	0.019051	0.3962	5	75003678	0.0016	1.60E-58	795430	BMI
rs2317299	C	T	-0.011	0.012357	0.5597	2	2.37E+08	0.0017	1.30E-09	677983	BMI
rs2325036	C	A	-0.018	0.030335	0.3845	3	85819412	0.0017	3.60E-27	790870	BMI
rs2357760	A	G	0.0145	-0.00952	0.6754	6	1.2E+08	0.0017	6.80E-17	791053	BMI
rs2361988	C	T	-0.016	0.049883	0.2539	16	398151	0.002	5.20E-15	690251	BMI
rs2365389	T	C	-0.017	-0.00107	0.4143	3	61236462	0.0017	1.30E-25	783625	BMI
rs2367112	G	T	-0.012	-0.05618	0.4919	5	64168193	0.0016	2.30E-13	794305	BMI
rs2423668	C	T	-0.011	0.039565	0.5505	20	12430673	0.0019	2.80E-08	527352	BMI
rs2425840	C	A	0.0119	-0.02661	0.4059	20	44904838	0.0018	1.60E-11	681680	BMI
rs2429150	C	A	0.0111	-0.03084	0.4164	12	2152655	0.0018	2.70E-10	686276	BMI
rs2479958	G	A	-0.015	0.02304	0.5075	13	1.12E+08	0.0018	1.50E-17	664268	BMI
rs2481665	C	T	-0.016	-0.04169	0.4408	1	62594677	0.0016	7.20E-23	795247	BMI

rs2543132	C	G	0.0146	0.02119	0.8134	8	15536311	0.0022	5.00E-11	688326	BMI
rs2600226	T	C	-0.012	-0.05225	0.6697	3	12928762	0.0019	3.70E-10	685285	BMI
rs2605603	A	G	-0.01	-0.04136	0.4887	11	93221105	0.0016	2.50E-10	790857	BMI
rs2608703	A	C	0.0142	0.015065	0.4546	12	41846769	0.0017	1.90E-16	686700	BMI
rs262130	T	C	0.0127	0.013208	0.1969	6	1.43E+08	0.0023	1.80E-08	679542	BMI
rs2693826	A	G	-0.014	-0.00072	0.4421	2	6160943	0.0017	2.00E-15	690808	BMI
rs2694047	G	A	0.0188	0.040755	0.747	8	1.17E+08	0.002	3.90E-21	690295	BMI
rs273504	G	A	0.0153	0.028379	0.4266	19	18215247	0.0018	4.40E-18	690672	BMI
rs2744974	T	C	0.0249	0.072178	0.338	6	34579431	0.0018	1.40E-45	789647	BMI
rs2791653	G	A	-0.014	0.036596	0.7577	1	11129848	0.0019	1.30E-13	795271	BMI
rs2820311	G	A	0.0235	0.034911	0.3369	1	2.02E+08	0.0018	4.10E-38	691876	BMI
rs2832283	A	G	0.0115	-0.01697	0.2208	21	30690558	0.002	5.80E-09	792324	BMI
rs2836964	C	T	-0.011	0.010329	0.3576	21	40631006	0.0018	1.30E-09	692353	BMI
rs2861683	C	A	-0.014	-0.03309	0.407	2	67836507	0.0017	1.30E-16	691163	BMI
rs2868975	A	G	-0.014	0.04021	0.178	3	1.17E+08	0.0023	2.20E-10	690613	BMI
rs287104	A	G	0.0115	-0.0783	0.6604	19	34290995	0.0017	4.40E-11	787307	BMI
rs2875762	C	G	0.0139	0.127307	0.2473	6	1.25E+08	0.002	1.20E-11	685199	BMI
rs2907948	A	G	-0.014	0.022319	0.2427	7	1.51E+08	0.0019	1.30E-13	794299	BMI
rs2931434	T	C	-0.01	0.025771	0.3168	5	73159098	0.0018	1.40E-08	690664	BMI
rs2943465	C	T	0.0248	-0.05094	0.9444	12	19265921	0.0039	2.00E-10	686547	BMI
rs294704	T	G	-0.011	0.027504	0.7239	5	1.53E+08	0.0019	4.00E-09	690036	BMI
rs3007105	T	C	0.0142	0.032162	0.4697	14	47367616	0.0017	1.10E-17	785488	BMI
rs326896	T	C	-0.013	0.024085	0.3925	4	1.13E+08	0.0018	2.80E-13	690324	BMI
rs331966	C	A	0.0112	-0.01504	0.3792	4	1.44E+08	0.0018	3.20E-10	687189	BMI
rs33500	T	C	-0.017	0.006239	0.8082	3	42427191	0.0022	4.30E-14	689500	BMI
rs339991	G	A	0.0124	-0.01702	0.5631	15	60913637	0.0018	1.20E-12	686476	BMI
rs349088	A	C	-0.013	-0.01603	0.4976	11	84814393	0.0017	1.80E-13	684401	BMI
rs355777	C	G	0.0153	0.073778	0.4106	3	1.54E+08	0.0017	1.40E-18	689978	BMI
rs3731695	C	T	0.0116	0.013138	0.5582	2	2.04E+08	0.0016	7.90E-13	793581	BMI
rs3732084	C	T	0.0107	0.00648	0.6139	2	2.07E+08	0.0018	1.10E-09	691763	BMI
rs3736485	G	A	-0.013	-0.00782	0.5443	15	51748610	0.0016	2.50E-16	790404	BMI
rs3749897	T	C	0.0122	0.028234	0.4172	6	42532102	0.0018	8.40E-12	638224	BMI
rs3754963	T	A	-0.012	-0.08174	0.2574	2	1.66E+08	0.002	3.30E-10	691535	BMI
rs3764835	A	G	-0.014	-0.00272	0.1528	2	1.6E+08	0.0024	3.10E-09	689505	BMI
rs3772882	A	C	0.0127	-0.0186	0.3661	3	81808602	0.0018	6.60E-13	691912	BMI
rs3800229	T	G	0.0175	0.071192	0.7123	6	1.09E+08	0.0018	1.40E-22	792474	BMI
rs3800637	C	T	0.0115	-0.01429	0.336	7	1.37E+08	0.0018	5.10E-10	682001	BMI
rs3806114	A	G	-0.011	-0.02433	0.6773	6	20482335	0.0018	3.40E-10	778855	BMI

rs3806572	A	G	-0.015	0.007131	0.2788	2	55238677	0.0019	1.60E-14	687646	BMI
rs3807645	A	G	-0.017	0.055682	0.221	7	77830091	0.0021	2.40E-15	683086	BMI
rs380857	A	C	-0.015	0.030058	0.8878	9	1.01E+08	0.0027	3.60E-08	691507	BMI
rs3814883	T	C	0.0232	-0.02567	0.4764	16	29994922	0.0017	1.10E-40	685519	BMI
rs3828783	A	G	-0.017	-0.01748	0.1809	6	33767727	0.0021	5.60E-15	792749	BMI
rs3829849	T	C	0.0098	0.05451	0.3589	9	1.29E+08	0.0017	5.90E-09	793851	BMI
rs38314	A	G	-0.012	-0.02059	0.4912	7	70067315	0.0017	4.70E-12	689782	BMI
rs3844598	G	A	0.0095	0.013019	0.521	5	1.41E+08	0.0017	3.80E-08	690704	BMI
rs3902951	G	T	0.0134	-0.00597	0.2455	14	69789755	0.002	7.00E-12	773819	BMI
rs3904244	A	T	0.0155	0.028203	0.1377	10	27361527	0.0025	4.30E-10	691180	BMI
rs391300	C	T	-0.012	-0.00222	0.6275	17	2216258	0.0017	3.10E-12	791120	BMI
rs3935648	G	C	-0.013	-0.00465	0.2328	17	79085335	0.0022	6.80E-09	629303	BMI
rs3977755	T	C	-0.014	-0.02939	0.2804	10	1.04E+08	0.0019	5.90E-13	731529	BMI
rs40067	A	G	-0.027	0.038786	0.1713	5	1.07E+08	0.0023	7.10E-30	681695	BMI
rs4012234	G	T	0.0141	-0.02132	0.5924	20	32553047	0.0018	9.90E-16	689653	BMI
rs4072917	A	G	0.0115	0.031018	0.4694	8	1.43E+08	0.0018	6.90E-11	684720	BMI
rs4148155	G	A	-0.019	-0.04048	0.1127	4	89054667	0.0026	5.00E-13	794889	BMI
rs4148866	T	C	0.0098	0.017656	0.4068	12	1.23E+08	0.0018	4.00E-08	676418	BMI
rs4237643	G	T	-0.022	-0.01966	0.6938	11	43648368	0.0019	4.30E-33	692491	BMI
rs427943	C	A	0.017	0.044777	0.5669	21	46570896	0.0017	7.30E-23	712095	BMI
rs429343	G	A	-0.015	-0.00034	0.5813	2	1.48E+08	0.0017	6.80E-18	689345	BMI
rs4307239	G	A	0.0115	0.027467	0.4578	7	24354300	0.0017	3.90E-11	687289	BMI
rs4358081	C	A	0.0097	-0.02486	0.4631	2	29100642	0.0017	1.50E-08	690038	BMI
rs4414033	A	G	0.0129	0.017139	0.627	1	1.56E+08	0.0018	1.40E-12	672697	BMI
rs4430672	C	T	-0.013	-0.02547	0.8004	14	63094407	0.0022	3.90E-09	691400	BMI
rs4482463	A	C	-0.033	-0.12567	0.9213	2	2.05E+08	0.0033	2.80E-23	635414	BMI
rs4516268	A	C	-0.022	0.07525	0.1925	17	1846831	0.0021	5.20E-25	786617	BMI
rs4518345	A	G	-0.012	0.012419	0.2842	5	27185904	0.0019	1.00E-09	688609	BMI
rs4556997	A	C	0.0197	0.030312	0.1349	2	1.01E+08	0.0024	6.90E-17	792972	BMI
rs4589691	G	C	0.0141	0.089742	0.1579	2	1.44E+08	0.0024	4.70E-09	688253	BMI
rs4639527	G	A	0.0172	0.005908	0.3012	2	416815	0.0019	3.30E-20	691706	BMI
rs4653017	T	C	0.0122	0.01476	0.6818	1	33776728	0.0018	4.50E-11	686378	BMI
rs4660443	T	C	0.0164	0.088613	0.2218	1	39591779	0.0021	6.80E-15	687234	BMI
rs4722398	T	C	0.0158	-0.01431	0.1336	7	3125220	0.0025	3.60E-10	692509	BMI
rs4740619	C	T	-0.019	-0.0153	0.4521	9	15634326	0.0016	2.30E-30	794491	BMI
rs4757144	A	G	0.0169	0.005195	0.5878	11	13331226	0.0018	5.60E-22	690082	BMI
rs4783830	A	G	-0.011	-0.05243	0.3074	16	54255346	0.0019	2.40E-08	675527	BMI
rs4786903	G	A	0.0125	0.031272	0.7368	16	6697104	0.002	3.50E-10	680139	BMI

rs4800191	C	G	0.0103	0.009905	0.6369	18	22461398	0.0017	2.50E-09	785353	BMI
rs4813619	T	G	-0.011	-0.04874	0.5101	20	2815715	0.0018	2.30E-09	622760	BMI
rs4818225	G	A	0.0117	0.014668	0.6606	21	42629895	0.0018	2.30E-10	688274	BMI
rs4842491	T	C	0.0098	-0.07053	0.7138	12	89905537	0.0018	4.00E-08	795312	BMI
rs4851029	G	T	0.0121	0.001565	0.5247	2	1.04E+08	0.0017	1.70E-12	689752	BMI
rs4858193	C	T	-0.013	0.031814	0.2779	3	20441050	0.0019	1.60E-11	686850	BMI
rs4864201	C	T	-0.014	-0.03679	0.6469	4	1.31E+08	0.0017	1.50E-16	795263	BMI
rs4880341	T	C	-0.012	-0.0148	0.5606	10	1.34E+08	0.0017	1.10E-11	689012	BMI
rs4906908	G	T	0.0103	0.060383	0.5253	15	27040082	0.0017	2.50E-09	691345	BMI
rs491711	C	A	-0.012	0.002171	0.316	11	28742220	0.0019	1.10E-09	685113	BMI
rs4929923	C	T	0.0181	-0.02518	0.6376	11	8639200	0.0017	7.20E-27	794933	BMI
rs4936175	C	T	0.0122	-0.02784	0.4445	11	1.33E+08	0.0017	1.40E-12	692569	BMI
rs4937870	G	A	-0.011	-0.0274	0.3172	11	1.13E+08	0.0019	8.80E-09	683154	BMI
rs4952843	G	A	-0.013	-0.01449	0.3807	2	46957845	0.0018	6.80E-14	692482	BMI
rs4954638	C	A	-0.012	0.00926	0.2492	2	1.37E+08	0.002	2.90E-09	689971	BMI
rs4968656	G	A	0.0116	-0.01903	0.3216	17	61616959	0.0019	8.20E-10	675153	BMI
rs4981693	A	G	0.0206	-0.05675	0.771	14	29680331	0.002	6.90E-24	689120	BMI
rs4986044	T	C	-0.016	-0.03721	0.4687	17	21261560	0.0016	3.30E-23	787219	BMI
rs538579	C	G	0.0137	0.08221	0.3228	3	62711674	0.0019	1.30E-13	688452	BMI
rs543874	G	A	0.0475	0.021851	0.1952	1	1.78E+08	0.002	1.20E-122	795504	BMI
rs559231	T	G	0.0135	0.047323	0.3956	18	39644247	0.0018	2.40E-14	685154	BMI
rs577525	C	T	0.0166	-0.03826	0.5676	10	99769388	0.0017	9.70E-22	690616	BMI
rs592483	T	C	-0.015	0.010317	0.5716	11	69445173	0.0017	2.00E-18	781871	BMI
rs6050446	G	A	0.0343	0.10229	0.97001	20	25195509	0.0047	4.40E-13	766287	BMI
rs6235	G	C	0.0175	0.058409	0.2702	5	95728898	0.0019	1.50E-19	691708	BMI
rs6265	T	C	-0.041	0.030213	0.1951	11	27679916	0.0021	1.00E-86	795458	BMI
rs6443750	C	T	0.0148	0.028939	0.8068	3	1.81E+08	0.0021	3.20E-12	776837	BMI
rs6448587	C	A	-0.017	-0.00057	0.1891	4	28561990	0.0023	2.30E-13	691097	BMI
rs645040	T	G	0.0171	-0.07973	0.7762	3	1.36E+08	0.002	2.50E-18	795579	BMI
rs6461115	G	A	-0.014	0.005298	0.2285	7	2103668	0.0019	1.20E-13	791735	BMI
rs6471941	A	G	0.0156	-0.00261	0.1684	8	62117973	0.0021	3.10E-13	793986	BMI
rs6500208	A	G	0.014	0.063201	0.2006	16	49011249	0.002	4.10E-12	781931	BMI
rs6512302	C	G	0.0142	0.021623	0.7511	20	62691550	0.002	2.10E-12	686053	BMI
rs6545714	A	G	-0.019	-0.04738	0.6139	2	59307725	0.0017	9.10E-31	793368	BMI
rs6556301	T	G	-0.011	0.031469	0.3596	5	1.77E+08	0.0018	4.10E-10	734744	BMI
rs6561943	T	C	0.0119	0.007336	0.2595	13	58356761	0.0019	4.20E-10	793951	BMI
rs657452	G	A	-0.019	-0.0311	0.6216	1	49589847	0.0017	7.20E-29	767846	BMI
rs6587552	G	A	-0.017	0.054889	0.7591	1	1.51E+08	0.002	1.60E-17	689723	BMI

rs6591407	A	C	-0.012	-0.02908	0.1861	11	56914157	0.0021	1.90E-08	794246	BMI
rs6593688	G	A	0.0137	0.024588	0.3733	1	96322205	0.0018	8.60E-15	691779	BMI
rs663129	A	G	0.0545	0.00274	0.2301	18	57838401	0.0019	1.60E-178	788948	BMI
rs6673081	C	T	-0.01	-0.04091	0.5534	1	1.55E+08	0.0018	1.80E-08	677818	BMI
rs6692586	G	A	-0.019	-0.03944	0.832	1	23299906	0.0023	1.10E-16	690921	BMI
rs6764533	A	G	0.0116	0.040146	0.359	3	1.96E+08	0.0018	1.40E-10	690832	BMI
rs6772756	G	A	-0.01	0.057431	0.3372	3	1.82E+08	0.0019	4.00E-08	681709	BMI
rs6785245	C	T	0.0132	0.017056	0.3969	3	82647990	0.0017	4.00E-14	692250	BMI
rs6804842	G	A	0.0156	-0.05886	0.572	3	25106437	0.0017	3.60E-21	789179	BMI
rs6841761	T	G	-0.013	-0.0136	0.5252	4	25423538	0.0016	6.40E-16	793477	BMI
rs685870	C	T	0.012	-0.01949	0.7035	11	64111928	0.0019	2.40E-10	688423	BMI
rs6985109	A	G	-0.018	0.026672	0.5338	8	10761585	0.0017	1.50E-26	793993	BMI
rs7024334	G	T	-0.014	-0.00373	0.7742	9	1.09E+08	0.002	3.10E-12	782431	BMI
rs7025938	G	C	0.0166	-0.02749	0.3187	9	1.03E+08	0.0019	3.70E-19	691581	BMI
rs7037266	A	C	-0.011	-0.02862	0.3739	9	6942940	0.0018	3.50E-10	691603	BMI
rs705217	G	T	-0.01	-0.0311	0.3652	1	34581472	0.0018	9.30E-09	688609	BMI
rs705704	A	G	-0.013	-0.01129	0.3304	12	56435412	0.0018	1.90E-13	743597	BMI
rs7084454	A	G	0.0193	-0.01542	0.335	10	21821274	0.0019	4.00E-25	678564	BMI
rs709400	G	A	-0.015	-0.01943	0.3818	14	1.04E+08	0.0017	4.60E-19	795379	BMI
rs7102454	C	T	0.0158	0.041436	0.3435	11	65594820	0.0018	2.40E-18	691134	BMI
rs7117238	A	G	-0.013	0.05123	0.168	11	78040259	0.0022	2.50E-09	788879	BMI
rs7124681	A	C	0.0263	-0.00629	0.4133	11	47529947	0.0016	3.20E-58	795474	BMI
rs7138803	A	G	0.03	0.015965	0.3772	12	50247468	0.0017	2.30E-71	795588	BMI
rs7144011	T	G	0.0282	0.050137	0.2136	14	79940383	0.002	5.20E-47	794117	BMI
rs7148846	G	T	0.0124	0.003203	0.1896	14	40133821	0.0022	2.20E-08	687940	BMI
rs7172627	G	A	0.0117	0.005053	0.4719	15	31877690	0.0017	1.10E-11	690458	BMI
rs7181498	C	T	-0.016	-0.01996	0.6309	15	95271404	0.0018	1.00E-19	690980	BMI
rs7196720	C	T	-0.013	-0.09986	0.5068	16	24534662	0.0017	7.30E-14	689863	BMI
rs7206608	G	C	0.0132	0.046284	0.3146	16	82872628	0.0019	1.30E-12	689058	BMI
rs7222349	A	G	0.0115	-0.0255	0.3441	17	42304644	0.0018	3.30E-10	692215	BMI
rs7239575	C	T	-0.02	-0.01056	0.4832	18	21120035	0.0017	7.40E-32	692313	BMI
rs7318817	T	C	-0.016	-0.04978	0.6071	13	28617708	0.0018	2.70E-18	691917	BMI
rs7334078	C	T	-0.012	0.045353	0.2882	13	99120484	0.0019	2.20E-10	688374	BMI
rs7358465	T	C	0.0103	0.022294	0.6781	11	89990280	0.0019	3.00E-08	686935	BMI
rs7488867	T	C	-0.02	0.003014	0.2639	12	1.04E+08	0.002	8.40E-24	635746	BMI
rs7498665	G	A	0.0271	0.005119	0.4038	16	28883241	0.0017	5.60E-60	790299	BMI
rs7535528	A	G	-0.015	0.048724	0.3741	1	2444414	0.0018	1.40E-16	632868	BMI
rs754635	G	C	0.0198	-0.103	0.8873	3	42305131	0.0027	2.20E-13	690346	BMI

rs7550711	T	C	0.0649	0.094007	0.03058	1	1.1E+08	0.005	3.20E-38	769184	BMI
rs7551507	T	C	-0.018	-0.02999	0.5633	1	74995225	0.0016	9.30E-30	794579	BMI
rs7557796	C	T	-0.016	-0.03326	0.6524	2	86766153	0.0018	2.30E-19	692414	BMI
rs756717	A	G	-0.015	0.001771	0.3973	16	72996162	0.0017	5.40E-18	771976	BMI
rs7599312	A	G	-0.019	-0.0388	0.2652	2	2.13E+08	0.0019	6.90E-24	780823	BMI
rs7615297	G	C	-0.015	0.063017	0.1465	3	1.56E+08	0.0024	5.70E-10	689710	BMI
rs7626079	T	C	0.011	0.016457	0.3434	3	66427259	0.0018	1.60E-09	692571	BMI
rs7637852	G	A	-0.014	-0.01085	0.6951	3	44041777	0.0019	1.70E-13	691815	BMI
rs7640424	T	C	-0.014	-0.03065	0.2969	3	1.08E+08	0.0018	2.30E-14	790612	BMI
rs765875	T	C	-0.012	0.033996	0.4808	6	1.43E+08	0.0017	3.00E-12	690961	BMI
rs7683836	A	G	-0.011	0.001024	0.5405	4	1.8E+08	0.0017	6.30E-11	686968	BMI
rs7685048	T	C	-0.01	0.077701	0.4654	4	95027784	0.0017	4.10E-09	692398	BMI
rs768840	A	G	0.0114	0.006933	0.4183	14	73143457	0.0018	2.00E-10	677485	BMI
rs769449	A	G	-0.025	-0.16136	0.1161	19	45410002	0.0027	2.30E-20	566857	BMI
rs7694732	G	A	-0.01	0.00464	0.4378	4	1.15E+08	0.0017	8.70E-09	690622	BMI
rs7703576	C	T	0.0103	-0.03259	0.2885	5	1.45E+08	0.0019	4.80E-08	690818	BMI
rs7704281	A	G	0.0271	-0.00895	0.04531	5	50591460	0.0041	6.50E-11	788585	BMI
rs7715256	T	G	-0.017	-0.02254	0.5781	5	1.54E+08	0.0016	2.20E-24	795302	BMI
rs7724675	A	G	-0.012	-0.01805	0.2238	5	1.3E+08	0.0021	9.50E-09	691968	BMI
rs7730004	T	C	0.0148	0.008414	0.6693	5	43191033	0.0018	9.10E-16	690164	BMI
rs7730898	A	G	0.0168	-0.00475	0.729	5	1.7E+08	0.0018	4.50E-20	792975	BMI
rs774246	G	A	0.0153	0.019838	0.1444	7	26990816	0.0025	5.40E-10	690818	BMI
rs7761673	A	T	-0.013	-0.0379	0.2058	6	70357368	0.0021	1.90E-09	691716	BMI
rs7780752	C	T	0.0139	0.000643	0.36	7	93241640	0.0018	1.00E-14	690830	BMI
rs7788008	A	G	-0.016	-0.02921	0.4445	7	1.13E+08	0.0017	1.10E-19	690410	BMI
rs7811342	C	T	-0.02	0.023829	0.1058	7	1.39E+08	0.0029	1.10E-11	676265	BMI
rs7819514	A	G	-0.011	0.067128	0.3216	8	93204442	0.0018	5.70E-09	684955	BMI
rs7826312	C	T	0.0104	-0.08783	0.5879	8	32400115	0.0017	4.90E-10	785343	BMI
rs7844647	C	T	-0.012	0.034007	0.2681	8	34503776	0.0018	2.80E-11	793703	BMI
rs7869771	C	A	-0.014	-0.00064	0.2647	9	94180627	0.0019	4.90E-13	679436	BMI
rs7871866	C	G	0.0187	-0.01614	0.1531	9	1.31E+08	0.0024	2.30E-14	683494	BMI
rs7899106	G	A	0.0331	-0.16126	0.04777	10	87410904	0.0037	1.00E-18	793689	BMI
rs7903146	T	C	-0.018	0.031409	0.2912	10	1.15E+08	0.0018	1.30E-23	795624	BMI
rs7925214	T	C	0.0147	-0.00536	0.5133	11	1.31E+08	0.0018	4.40E-17	677603	BMI
rs7970953	A	G	0.0135	0.066084	0.29	12	24075508	0.0018	9.80E-14	788417	BMI
rs7983065	T	C	-0.015	-0.04847	0.4503	13	33380786	0.0017	8.90E-18	690924	BMI
rs7998796	G	A	0.0105	-0.00447	0.3373	13	81020036	0.0018	1.10E-08	689430	BMI
rs8027205	G	C	-0.011	-0.03837	0.3967	15	98280959	0.0018	1.40E-09	685725	BMI

rs8036040	A	C	0.0109	-0.01638	0.4932	15	36402716	0.0017	2.70E-10	691068	BMI
rs8047395	A	G	0.0642	0.01946	0.5061	16	53798523	0.0017	1.00E-200	788856	BMI
rs806600	G	A	-0.01	-0.00912	0.475	5	1.73E+08	0.0017	3.30E-08	691791	BMI
rs8071182	A	G	0.0133	0.023946	0.1735	17	55336155	0.0022	2.10E-09	771437	BMI
rs8090983	G	A	0.0118	-0.06319	0.3314	18	52586691	0.0018	2.00E-10	682470	BMI
rs8097672	T	A	0.02	-0.01782	0.1528	18	1839601	0.0025	8.40E-16	686063	BMI
rs8097783	A	G	-0.039	-0.03416	0.07554	18	58051294	0.0031	7.20E-36	795408	BMI
rs8123881	G	A	0.0196	-0.0088	0.1299	20	15819495	0.0024	4.40E-16	793018	BMI
rs8181823	C	A	0.0127	-0.0951	0.7614	13	65477940	0.002	4.10E-10	691345	BMI
rs818524	C	T	0.0106	0.040535	0.6939	1	85201228	0.0019	3.40E-08	670078	BMI
rs8192675	C	T	0.0152	-0.031	0.2888	3	1.71E+08	0.0018	1.40E-17	795515	BMI
rs825688	T	C	-0.01	-0.00336	0.456	16	73595718	0.0017	4.70E-08	686144	BMI
rs845084	A	G	0.014	0.009889	0.2678	10	1.25E+08	0.002	1.30E-12	685413	BMI
rs852056	C	T	-0.013	-0.04919	0.7584	20	17102860	0.002	1.80E-10	691874	BMI
rs865809	G	A	-0.013	-0.09001	0.7678	3	1.84E+08	0.002	5.40E-10	689186	BMI
rs872281	T	C	-0.015	0.050219	0.1728	14	40834177	0.0023	4.70E-11	685310	BMI
rs876605	G	A	-0.011	0.007029	0.7352	5	77801359	0.002	3.40E-08	692586	BMI
rs879620	T	C	0.0231	0.044292	0.6179	16	4015729	0.0018	5.30E-38	688377	BMI
rs889398	T	C	-0.02	0.008948	0.4247	16	69556715	0.0016	1.30E-32	789694	BMI
rs895330	G	C	-0.02	-0.02366	0.1924	19	4060707	0.0023	5.50E-19	684271	BMI
rs901630	T	C	-0.015	0.013424	0.3973	6	98539519	0.0017	1.90E-18	794597	BMI
rs902695	A	G	-0.01	-0.02343	0.4798	2	1.14E+08	0.0017	2.20E-09	678635	BMI
rs9294260	A	G	0.0147	-0.06081	0.4731	6	83433228	0.0016	1.80E-19	783533	BMI
rs9300422	G	A	-0.01	-0.03278	0.6903	13	98223320	0.0018	4.00E-09	795011	BMI
rs930295	C	A	-0.021	0.031368	0.8417	2	50233352	0.0023	1.00E-19	690522	BMI
rs9304665	A	T	0.0229	-0.00262	0.7633	19	47602577	0.002	2.90E-29	689470	BMI
rs934224	T	C	0.0107	-0.00613	0.7399	2	16613889	0.002	4.70E-08	692568	BMI
rs9362662	G	A	-0.011	0.02218	0.5201	6	90296588	0.0017	1.20E-10	683953	BMI
rs9367368	C	T	-0.012	0.010028	0.3033	6	13189275	0.0018	1.00E-11	786723	BMI
rs9375702	T	C	-0.012	-0.01313	0.705	6	1.3E+08	0.0019	7.90E-10	690564	BMI
rs9379827	A	C	-0.013	-0.02302	0.2409	6	26153335	0.0019	6.90E-12	795072	BMI
rs9408882	A	G	-0.009	0.032208	0.4594	9	1.19E+08	0.0016	1.30E-08	794283	BMI
rs946824	C	T	-0.021	0.051566	0.859	1	2.44E+08	0.0026	1.10E-15	689849	BMI
rs947612	A	G	-0.012	0.012468	0.7516	6	73738661	0.002	5.60E-09	692596	BMI
rs9522285	A	G	0.0127	0.01221	0.4143	13	1.12E+08	0.0017	2.50E-13	690681	BMI
rs9538162	C	T	-0.016	-0.01056	0.4138	13	59265043	0.0018	4.80E-19	690345	BMI
rs9547153	G	A	0.0098	-0.02303	0.3839	13	85903717	0.0017	8.70E-09	775400	BMI
rs9571687	A	C	-0.013	-0.07406	0.329	13	67472713	0.0018	2.80E-12	690974	BMI

rs962273	C	T	0.0137	-0.03497	0.7057	17	46978353	0.0019	2.60E-13	692594	BMI
rs9650755	G	A	0.0154	-0.05371	0.2664	9	96484342	0.002	2.80E-15	691183	BMI
rs9688431	C	T	-0.023	-0.00689	0.06034	6	73922654	0.0035	2.40E-11	789356	BMI
rs977747	G	T	-0.017	-0.03404	0.5949	1	47684677	0.0017	1.30E-24	793546	BMI
rs9783858	T	C	0.0091	0.052352	0.5191	18	42534584	0.0017	3.30E-08	770874	BMI
rs9806742	A	G	0.0208	-0.03961	0.8826	15	73051219	0.0026	1.40E-15	692509	BMI
rs9816226	T	A	0.0323	0.0465	0.8199	3	1.86E+08	0.0021	1.60E-52	778333	BMI
rs9845966	G	T	-0.011	0.012413	0.5479	3	13433158	0.0017	2.50E-10	778076	BMI
rs987237	G	A	0.0409	-0.05002	0.1803	6	50803050	0.0021	9.30E-84	795612	BMI
rs9926784	C	T	-0.026	-0.01569	0.1822	16	19941968	0.0021	9.90E-35	789617	BMI
rs9927848	A	C	-0.012	-0.01679	0.7326	16	23833071	0.002	6.40E-10	687060	BMI
rs9951619	G	T	0.0156	-0.07734	0.7643	18	56882326	0.002	1.40E-15	772643	BMI
rs998732	G	A	-0.017	-0.02449	0.1578	19	19378671	0.0022	2.00E-14	793852	BMI
rs9989141	T	C	0.0162	0.056574	0.6387	14	94006257	0.0017	3.60E-21	752768	BMI
rs999889	A	G	-0.011	0.006019	0.2818	10	84279949	0.0019	1.40E-08	690572	BMI
rs10132280	A	C	-0.022	-0.10245	0.3333	14	25928179	0.0037	2.20E-09	231985	WC
rs10767658	G	C	-0.031	0.013797	0.6417	11	27672252	0.0037	3.30E-17	225754	WC
rs10840100	G	A	0.02	-0.0179	0.725	11	8669437	0.0035	5.40E-09	232029	WC
rs10938397	G	A	0.032	0.01222	0.4333	4	45182527	0.0035	6.10E-20	231679	WC
rs10968576	G	A	0.025	0.025186	0.2917	9	28414339	0.0036	1.20E-11	232050	WC
rs11165623	A	G	0.02	0.002434	0.4833	1	96893000	0.0034	5.20E-09	232057	WC
rs12429545	A	G	0.031	-0.03578	0.1	13	54102206	0.0052	2.50E-09	222998	WC
rs1516725	C	T	0.031	0.083459	0.9083	3	1.86E+08	0.0051	1.70E-09	230610	WC
rs16894959	C	T	0.026	0.101408	0.1	6	34825662	0.0048	3.40E-08	230687	WC
rs16996700	C	T	-0.023	0.05759	0.3	20	50981945	0.0037	1.50E-09	231903	WC
rs17066856	C	T	-0.037	-0.05933	0.1333	18	58049656	0.006	9.00E-10	230221	WC
rs2112347	G	T	-0.025	0.009348	0.375	5	75015242	0.0035	3.20E-13	232028	WC
rs2287019	T	C	-0.035	-0.01988	0.15	19	46202172	0.0046	1.70E-14	217525	WC
rs2293576	A	G	-0.022	0.065995	0.3667	11	47434986	0.0036	9.40E-10	226024	WC
rs2325036	C	A	-0.023	0.030335	0.4083	3	85819412	0.0035	2.10E-11	232048	WC
rs2489623	C	A	0.019	0.038509	0.5583	6	1.27E+08	0.0034	3.40E-08	231857	WC
rs2531992	G	A	0.028	0.005342	0.8333	16	4021734	0.0048	3.00E-09	232034	WC
rs2820292	C	A	0.019	0.011185	0.5083	1	2.02E+08	0.0034	2.40E-08	231899	WC
rs3127553	A	G	-0.023	-0.05887	0.6333	1	49438005	0.0035	1.60E-10	231815	WC
rs3849570	A	C	0.021	-0.03048	0.3667	3	81792112	0.0038	2.20E-08	196103	WC
rs4776970	T	A	-0.02	-0.0295	0.3417	15	68080886	0.0035	2.30E-08	230989	WC
rs6163	A	C	0.019	0.016076	0.3917	10	1.05E+08	0.0035	3.70E-08	225708	WC
rs633715	C	T	0.043	0.029835	0.2667	1	1.78E+08	0.0043	3.30E-23	218883	WC

rs6440003	A	G	0.021	0.003141	0.4833	3	1.41E+08	0.0034	2.90E-10	231985	WC
rs6545714	A	G	-0.022	-0.04738	0.625	2	59307725	0.0035	1.90E-10	232046	WC
rs6567160	C	T	0.048	-0.00134	0.2833	18	57829135	0.004	2.60E-33	231894	WC
rs6755502	C	T	0.051	0.0873	0.875	2	635721	0.0045	2.00E-30	231849	WC
rs7138803	A	G	0.028	0.015965	0.4417	12	50247468	0.0035	1.60E-15	232059	WC
rs7144011	T	G	0.033	0.050137	0.275	14	79940383	0.0041	9.40E-16	232007	WC
rs7239883	A	G	-0.021	-0.02678	0.6833	18	40147671	0.0035	2.30E-09	231731	WC
rs749671	A	G	-0.019	0.029413	0.375	16	31088347	0.0035	3.20E-08	232060	WC
rs7498665	G	A	0.034	0.005119	0.3583	16	28883241	0.0035	1.40E-22	230193	WC
rs7531118	C	T	0.027	0.034094	0.6083	1	72837239	0.0035	1.50E-14	231912	WC
rs7550711	T	C	0.058	0.094007	0.0339	1	1.1E+08	0.0098	3.40E-09	212149	WC
rs7903146	T	C	-0.022	0.031409	0.25	10	1.15E+08	0.0037	3.90E-09	232078	WC
rs806794	G	A	-0.022	0.009255	0.275	6	26200677	0.0037	2.10E-09	225694	WC
rs929641	G	A	-0.021	-0.02328	0.3833	2	58792377	0.0034	1.20E-09	231976	WC
rs9400239	C	T	0.024	0.072251	0.7	6	1.09E+08	0.0036	1.90E-11	232015	WC
rs943005	T	C	0.039	-0.05685	0.1	6	50865820	0.0044	7.20E-19	232080	WC
rs10245353	A	C	0.035	0.0017	0.1833	7	25858614	0.0043	8.40E-16	210008	WHR
rs10804591	A	C	0.024	0.013031	0.85	3	1.29E+08	0.0042	6.60E-09	209921	WHR
rs10842707	T	C	0.032	0.049206	0.1667	12	26471364	0.004	4.40E-16	210023	WHR
rs10991437	A	C	0.031	0.020952	0.1	9	1.08E+08	0.0054	1.00E-08	209941	WHR
rs11231693	A	G	0.041	-0.0285	0.0417	11	63862612	0.0075	4.50E-08	198072	WHR
rs1128249	T	G	-0.028	-0.03099	0.4417	2	1.66E+08	0.0035	2.00E-15	209414	WHR
rs12143789	C	G	0.024	0.036285	0.1583	1	1.19E+08	0.0042	7.60E-09	209874	WHR
rs12608504	G	A	-0.022	0.005944	0.6583	19	18389135	0.0036	8.80E-10	209990	WHR
rs12679556	G	T	0.027	-0.01578	0.2083	8	72514228	0.004	2.10E-11	203826	WHR
rs1294410	C	T	0.031	-0.02735	0.625	6	6738752	0.0035	2.00E-18	209830	WHR
rs1385167	G	A	0.029	-0.07421	0.1417	2	66200648	0.0049	1.80E-09	206619	WHR
rs1440372	C	T	0.024	0.067705	0.7417	15	67033151	0.0038	1.10E-10	207447	WHR
rs1569135	G	A	-0.021	0.023446	0.4667	2	1.88E+08	0.0034	5.60E-10	209906	WHR
rs17451107	C	T	-0.026	-0.02729	0.375	3	1.57E+08	0.0036	1.10E-12	207795	WHR
rs17819328	G	T	0.021	-0.02228	0.45	3	12489342	0.0035	2.40E-09	208809	WHR
rs1936805	T	C	0.042	0.040176	0.55	6	1.27E+08	0.0034	3.60E-35	209859	WHR
rs2071449	A	C	0.028	0.01071	0.325	12	54428011	0.0036	3.00E-14	206953	WHR
rs2294239	G	A	-0.025	-0.03015	0.45	22	29449477	0.0035	7.20E-13	209454	WHR
rs2645294	T	C	0.031	-0.03464	0.5345	1	1.2E+08	0.0035	1.70E-19	209808	WHR
rs2820443	C	T	-0.035	0.005602	0.3	1	2.2E+08	0.0037	5.30E-21	209975	WHR
rs303084	A	G	0.023	0.034866	0.7833	4	1.24E+08	0.0042	3.90E-08	209941	WHR
rs4081724	A	G	-0.035	-0.08673	0.15	19	33824946	0.0051	7.40E-12	207418	WHR

rs459193	G	A	-0.026	-0.04188	0.7833	5	55806751	0.0038	1.60E-11	209952	WHR
rs4646404	A	G	-0.027	0.025469	0.375	17	17420199	0.0039	1.40E-11	198196	WHR
rs4765219	A	C	-0.028	0.046694	0.375	12	1.24E+08	0.0036	1.60E-15	209807	WHR
rs6090583	G	A	-0.022	-0.02182	0.5667	20	45558831	0.0034	6.20E-11	209435	WHR
rs6772129	G	A	-0.035	0.026194	0.2417	3	64700425	0.0037	3.40E-21	210055	WHR
rs714515	A	G	-0.027	0.008502	0.5417	1	1.72E+08	0.0034	4.40E-15	203401	WHR
rs7705502	A	G	0.027	0.072841	0.2917	5	1.73E+08	0.0036	4.70E-14	209827	WHR
rs8030605	A	G	0.03	0.076608	0.1583	15	56504598	0.0053	8.80E-09	208374	WHR
rs878639	G	A	-0.021	-0.05346	0.3583	20	33894463	0.0035	5.10E-09	207490	WHR
rs905938	C	T	-0.025	0.025433	0.325	1	1.55E+08	0.004	7.30E-10	207867	WHR
rs979012	C	T	-0.027	0.006341	0.6417	20	6623374	0.0036	3.30E-14	209941	WHR
rs998584	A	C	0.043	-0.04306	0.475	6	43757896	0.0038	1.10E-29	189620	WHR
rs9991328	T	C	0.018	-0.03956	0.4833	4	89713121	0.0034	4.50E-08	209925	WHR
